# Liquid Alkaline Water Electrolyzers: Comparing Performance across Design, Operation, and End-of-Life Scenarios

**DOI:** 10.1021/acs.est.5c07500

**Published:** 2025-10-10

**Authors:** Mohammed T. Zaki, Colby Smith, Alex Badgett, Hanna M. Breunig

**Affiliations:** † Energy Analysis and Environmental Impacts Division, Energy Technology Area, 1666 Lawrence Berkeley National Laboratory, Berkeley, California 94720, United States; ‡ Strategic Energy Analysis Center, National Renewable Energy Laboratory, Golden, Colorado 80401, United States

**Keywords:** life cycle assessment, hydrogen production, hydrogen leakage, balance of plant, stack recycling

## Abstract

Liquid alkaline water electrolysis (LAWE) is a demonstrated technology for hydrogen production, yet a comprehensive life cycle assessment (LCA) of their deployment is lacking. Research leading to improvements to the core component, the electrochemical stack, along with auxiliary system materials and dynamic operation of stacks from variable electricity supply offers new data that allows for detailed modeling and evaluation. Here, we present an LCA of two facility designs based on the current state-of-the-art stack and an advanced stack with zero-gap between electrodes, and capture dynamic electricity use from solar, wind, and hybrid sources and stack recycling strategies. We present life cycle impact factors characterizing the production of 1 kg of hydrogen across 12 environmental, human health, and resource impact categories (TRACI and ReCiPe) in the contiguous United States. As expected, the source of electricity will drive impacts (e.g., 83–94% of carbon intensity); however, we find that operating using wind electricity can lower hydrogen leakage and the overall carbon intensity (1.03 kgCO_2_e/kgH_2_) relative to solar electricity (2.57 kgCO_2_e/kgH_2_) at matched 1:1 capacity between LAWE and the electricity source. The deployment of the advanced design and stack recycling lowers impacts across all life cycle stages. We highlight opportunities to further reduce potential impacts, including the balance of plant materials and operation cycles associated with the use of variable wind and solar electricity that result in hydrogen leakage.

## Graphical abstract

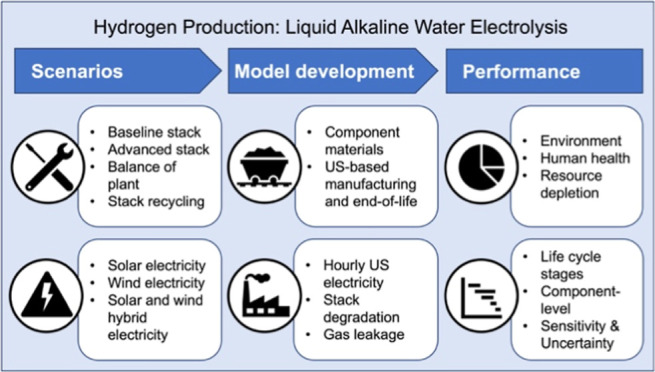

## 1 Introduction

Liquid alkaline water electrolyzers (LAWEs) are a commercial, scalable, and cost-effective technology used to generate ammonia, oxygen, and hydrogen (H_2_) at low temperatures (50–100 °C) generally without the use of noble metals.^1–3^ New investments in LAWE have led to stack designs with potential to produce H_2_ at improved energy and material efficiencies using electricity from variable power sources such as wind and solar.^4–7^ One such design employs a “zero-gap” configuration, while current state-of-the-art designs have been in use for decades.^4,8,9^ The LAWE stack comprises electrodes to facilitate electrochemical reactions, membrane to transport ions, gaskets to avert gas escape, and frame and plates to provide separation and electrical conduction in the system.^4^ In a “zero-gap” stack design, the electrodes are in direct contact with the membrane, which eliminates the electrolyte gap typically present in the current state-of-the-art stack designs.^1,2^ The elimination of the electrolyte gap in the “zero-gap” design reduces ohmic resistance, which improves energy efficiency by enhancing ion transport. It also minimizes the amount of materials needed to construct the plates by reducing the surface area for electrical conduction.^1,4,10^ An improved understanding of the life cycle impacts resulting from LAWE stack design and usage can inform scaled-up deployment of hydrogen production by indicating hotspots in materials and energy use.

Life Cycle Assessments (LCAs) estimate potential resource, human health, and environmental impacts of a product throughout a given product cycle (e.g., cradle-to-grave or well-to-gate). Life cycle stages include raw materials acquisition, product manufacturing, product Operation and Maintenance (O&M), and waste materials treatment (disposal or recycling) at the end of product life.^3,11^ The robustness of an LCA depends on the quality of life cycle inventory data and model assumptions used to represent each life cycle stage. Existing literature on LAWE LCAs tends to study the impacts associated with the material acquisition and manufacturing of stacks (i.e., embodied impacts) using relatively general material and energy balances as inputs for the life cycle inventory analysis, without providing further information on the fundamentals behind these values and guidance on the end-of-life treatment of waste materials (Section S1 and Table S1).^3,12–20^ We address these gaps in order to: (1) recommend strategies for improving materials of not only stacks but also auxiliary equipment such as Balance of Plant (BoP), (2) study the effects of dynamic operation of LAWEs coupled with variable power generation on electricity usage, cell degradation, and H_2_ gas leakage, and (3) formulate treatment strategies of waste materials.^21–24^ Further, we report model uncertainties through Monte Carlo simulations that arise from the nature of available data and model assumptions at the various life cycle stages, thereby allowing for a robust estimation of life cycle impacts that can lead to economic considerations for deployment. By addressing these gaps, this analysis can inform the scale-up of hydrogen production. This is specifically important in the US, where investments are in place to support activities on local manufacturing and recycling of LAWEs and to connect these systems with variable electricity sources for H_2_ production.^25,26^

In this study, we present an LCA of the current state-of-the-art (hereafter referred to as “baseline”) and “zero-gap” (hereafter referred to as “advanced”) stack designs as well as BoP across all of the life cycle stages (cradle-to-grave) by integrating available inventory data and models collected from the scientific literature and building US-based supply chain process flows in OpenLCA 2.0.4 and ecoinvent 3.10. The effects of variable electricity supply on H_2_ production and associated operational nuances such as cell degradation and H_2_ gas leakage are captured and modeled using hourly wind, solar, and hybrid (wind and solar) power generation data. Although the scope of our study is focused on H_2_ production, we would like to note that the requirements of an end user may influence stack operation but are outside the scope of this analysis. Overall, the outcome of this study has the potential to provide important insights to guide the deployment of LAWEs through improved material and energy-use efficiencies.

## 2 Materials and Methods

Our LCA method follows the ISO 14040/14044 guideline by including four stages of analysis (goal and scope definition, life cycle inventory analysis, life cycle impact assessment methods, and result interpretation) as discussed below.^27,28^

### 2.1 Goal and Scope Definition

The goal of this study was to assess the cradle-to-grave environmental impacts of a LAWE for H_2_ production using wind and solar as the electricity source across various stack designs, dynamic operations, and recycling strategies in the US. The system boundary of our LCA includes raw materials acquisition (mining, processing, and transporting), product manufacturing and transportation, and waste materials treatment of the baseline and advanced stacks along with BoP components of a LAWE (Figure 1). The system boundary included solar panels and wind turbines for the electricity supply, ion exchangers for the water supply, and chemical factories for the supply of electrolytes (potassium hydroxide (KOH)) and nitrogen for maintenance. To identify effective recycling strategies for waste materials, the system boundary expanded to include the connection from the recycling plant to product manufacturing based on the viability of replacing raw materials with recycled materials and improving material efficiency (Figure 1). We chose 1 kg of H_2_ produced as the functional unit for the LCA based on past studies, where it was assumed that a 48 MW stack was directly connected to a 48 MW electricity source across all scenarios (Table S1). The stack design pressure was 1.3 bar absolute, operating temperature was 80 °C, liquid electrolyte solution was 7 M aqueous KOH (∼30 wt %), and purity was 99.99 mol % H_2_.^4,20^

**Figure 1:**
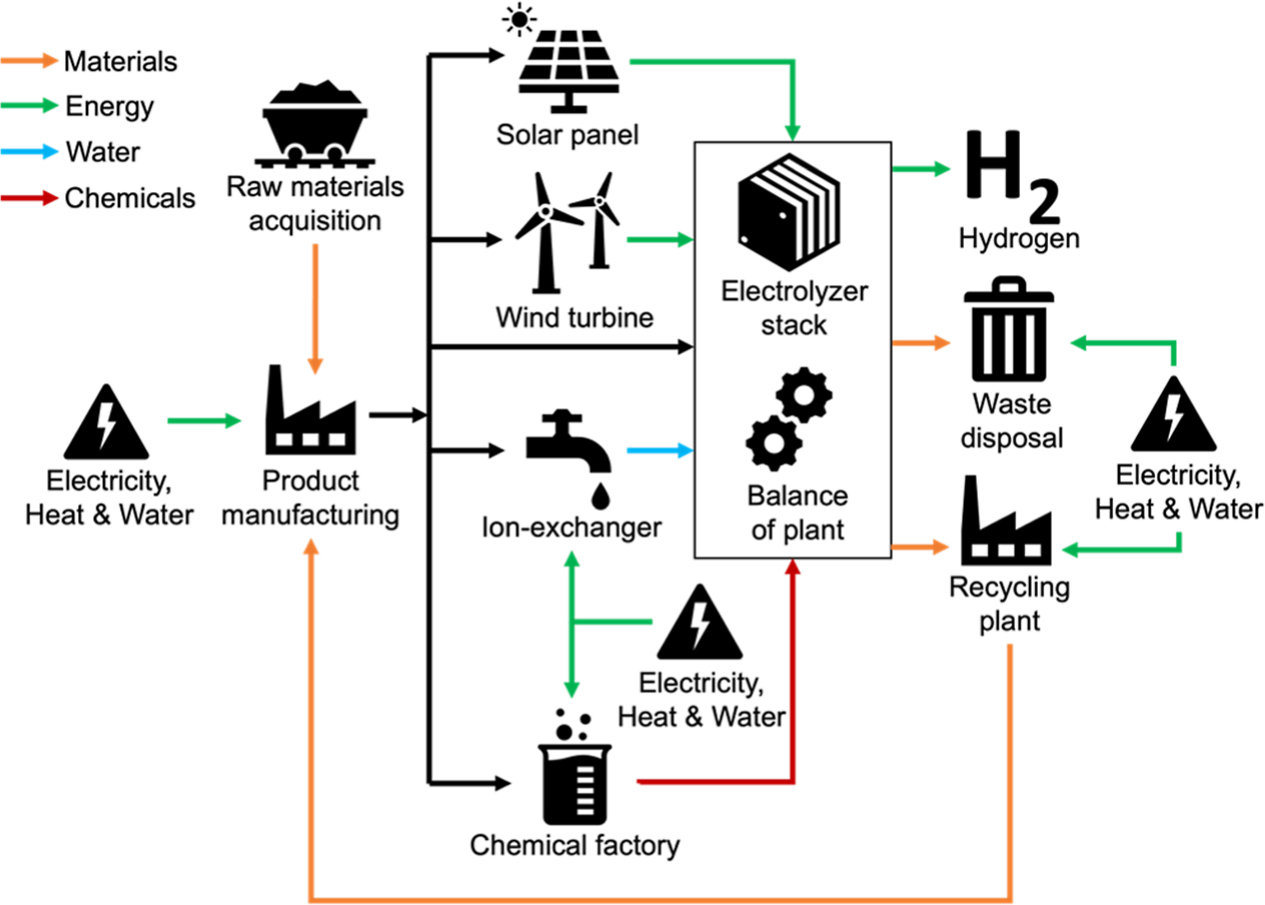
System boundary for the cradle-to-grave LCA of a LAWE system.

### 2.2 Life Cycle Inventory Analysis

A robust and representative materials inventory is essential to explicitly understand the life cycle impacts of acquiring and disposing different materials and resources to manufacture and operate a LAWE system. To achieve this, we built our life cycle inventory by collecting secondary data on material weights and respective key components and subcomponents from 10 peer-reviewed LCA studies in scientific literature published during 2017–2024 (Tables S1 and S2), integrating an operational model based on hourly variable electricity supply from solar, wind, and hybrid (solar and wind) sources in three case study locations in the US, and modifying global or rest-of-the-world market unit processes into US-representative market unit processes, as described below.

#### 2.2.1 Materials and Components

Our inventory consisted of the flow of weights for the different materials, as shown in Figure 2 and further described in Tables S2 and S3. Within the stack component, the largest contribution of materials came from steel, which is mainly used in the manufacturing of the different subcomponents: bipolar plates, end plates, electrodes (anode and cathode), and frame (Figure 2b,c). However, in the advanced stack design, the use of steel in the bipolar plates and end plates is 93% less than the baseline stack design. Among other major materials, nickel is used in the bipolar plates, end plates, and electrodes, which is used 77% less in the advanced stack design than the baseline design (Figure 2b,c). It should be noted that material weights of steel and nickel in bipolar and end plates were not considered in most studies except for the two recent studies that compared baseline and advanced stack designs.^18,19^ The substantial reductions in the amount of steel and nickel in the bipolar plates and end plates are because of the compact assembly of the electrodes and membrane in the advanced stack design compared to the baseline design. Specifically, for reduced electrical resistance in the advanced design, electrodes and membranes are tightly assembled unlike the baseline design where electrodes and membranes remain at greater distances in an open assembly, where thinner Zirfon (made of Zirconium oxide) is used as the membrane in the advanced design (200 μm instead of the 500 μm in the baseline design).^4,19,29^ Additionally, to achieve a greater electrochemically active surface area, the advanced stack electrodes use nickel mesh in the anode and nickel mesh with Raney nickel coating in the cathode, unlike the nickel-plated steel electrodes in the baseline stack. This also contributes to the reduced use of steel and nickel. The gasket is composed of plastics and rubber such as polyethylene, tetrafluoroethylene, and copolymers (Figure 2b,c).^4,19^ We used energy requirements for the manufacturing of both the baseline and advanced stack from a previous US-based study (Table S4).^19^

**Figure 2:**
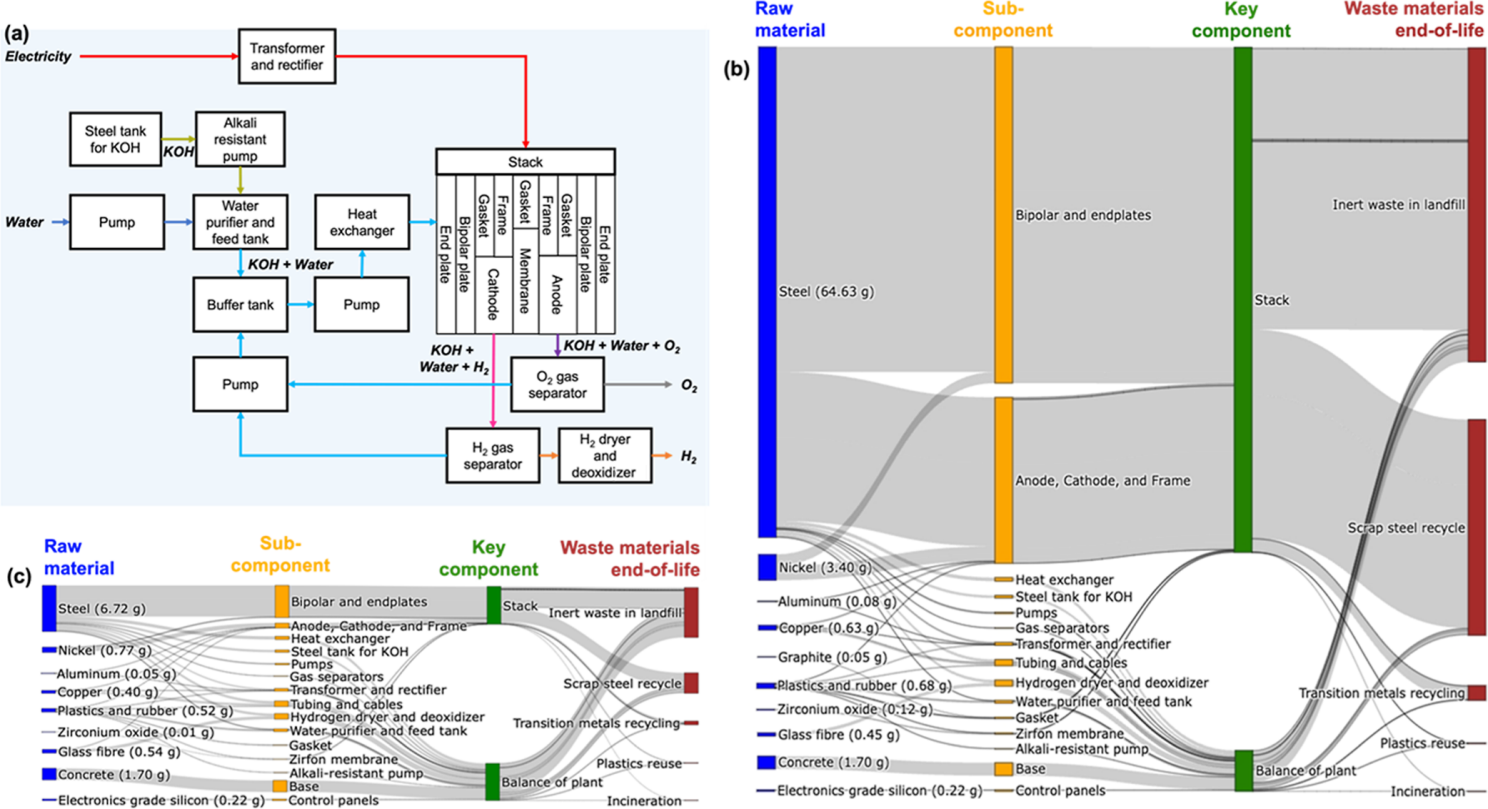
(a) A general representation of a LAWE with different subcomponents and components based on the literature review. Sankey diagram showing the flow of materials (median weights per kg H_2_) from acquisition to subcomponents to key components to waste material end-of-life treatment for the (b) baseline and (c) advanced stack designs with balance of plant (notations: KOH = potassium hydroxide, H_2_ = hydrogen, and O_2_ = oxygen).

The BoP subcomponents included water purifier, pumps, tanks, heat exchanger, transformer, rectifier, and gas separators (Figure 2a), which are mainly composed of steel (Figure 2b,c). Other notable BoP subcomponents are the control panel composed of electronics-grade silicon, tubing and cables composed of copper and tube insulation, and H_2_ dryer and deoxidizer composed of glass fiber (Figure 2b,c). We assume that the base of the LAWE is composed of reinforced concrete blocks made of Portland cement (Figure 2b,c). Energy requirements for the manufacturing of BoP subcomponents were based on processes obtained from a previous study that included sheet rolling for steel and aluminum, injection molding for plastics, and wire drawing for copper (Table S5).^15^

For the end-of-life treatment of waste materials after decommissioning, we leveraged information from a previous study (which partially utilized ecoinvent 3.10 for inventory analysis based on European market processes) to set the flow of specific materials to specific end-of-life treatment inventory processes such as recycling, landfilling, and incineration (Tables S2 and S3).^16^ To calculate the weights of material flow from the stack and BoP to their respective end-of-life treatment processes (based on most recent landfilling, recycling, and incineration rates; Figure 2), U.S. Geographic Survey’s Minerals Yearbook for Metals and Minerals^30^ and US Environmental Protection Agency’s Plastics Waste Management^31^ were used (Tables S2 and S3).

#### 2.2.2 Operation and Maintenance

Our O&M inventory included an operational model to estimate impacts from electricity use and H_2_ gas leakage based on hourly electricity supply, stack lifetime, cell degradation, and the shutdown and start-up of the system. For the operational model, we assumed that a 48 MW stack was directly connected to a 48 MW electricity source representing three case study locations in the US: wind turbines in Casper, Wyoming, solar panels in Daggett, California, and hybrid solar panels and wind turbines in Amarillo, Texas (Figure 3). These electricity outputs for the wind turbines and solar panels at the three case study locations were obtained for 2023 through simulations from the National Renewable Energy Laboratory PySAM (Python System Advisor Model) modeling platform.^32^ Hourly electricity generation profiles at the three case study locations illustrate the distribution of generation intensity (in MW) by day and hour over one year, with darker shades indicating higher output and lighter shades representing periods of low generation. Wind generation in Casper, Wyoming, is intermittent and can occur at any hour, showing variable patterns throughout the year, although there is relatively lower output during summer (Figure 3a). Solar generation in Daggett, California, peaks around midday and is zero at night, with higher output in summer (Figure 3b). The hybrid profile in Amarillo, Texas, combines solar and wind, reducing variability and extending generation across more hours, thereby enhancing reliability (Figure 3c).

**Figure 3:**
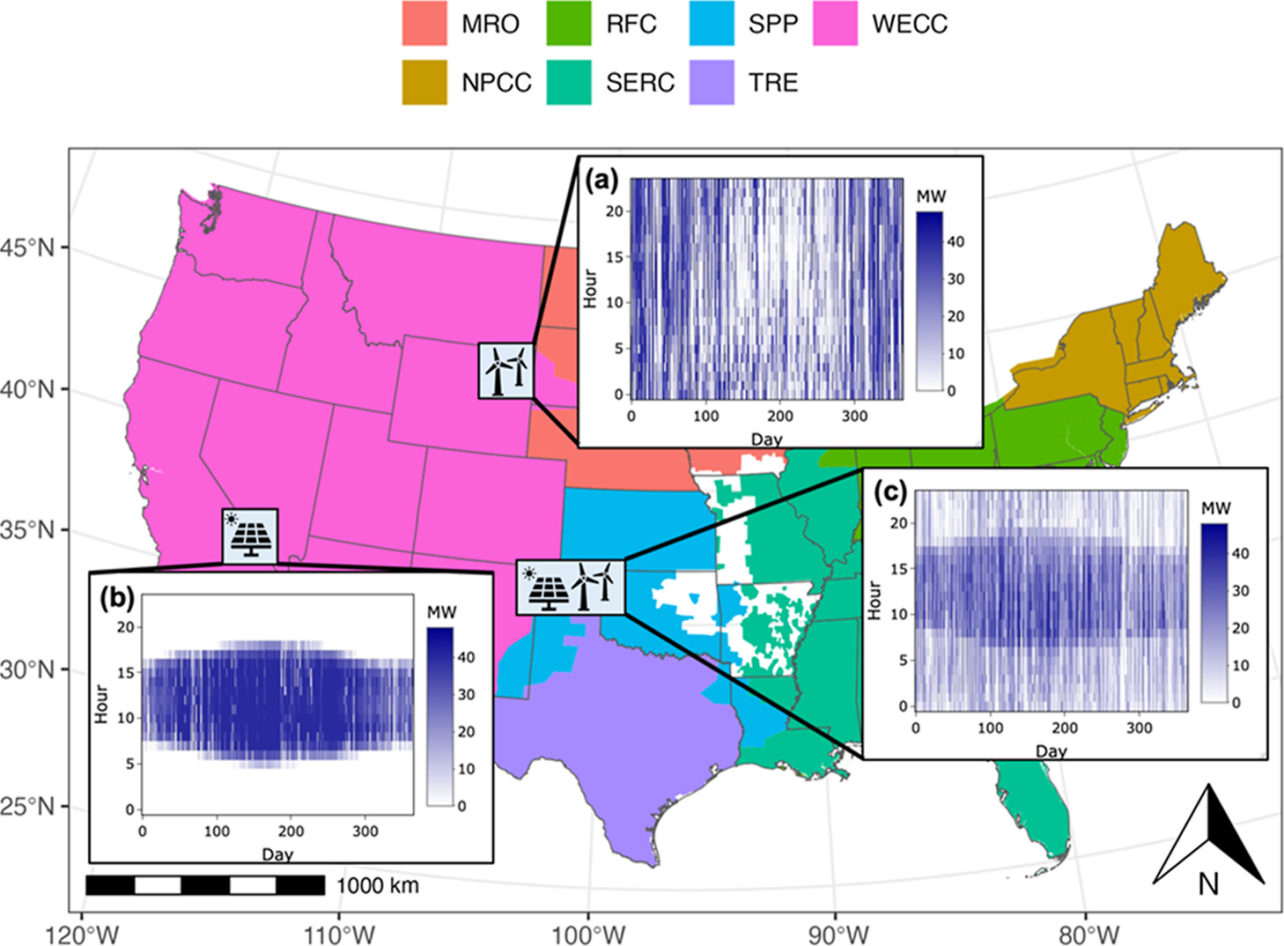
Locations and seasonal profiles of variable power generation output at (a) 48 MW wind turbines in Casper, Wyoming, (b) 48 MW solar panels in Daggett, California, and (c) 48 MW hybrid solar panels and wind turbines in Amarillo, Texas. The projection of the map is based in the US Geological Survey Contiguous Albers Equal Area, the US States boundaries were collected from US Census Bureau cartographic boundary files, and the North American Reliability Corporation region boundaries were collected from US Energy Information Administration Atlas (notations: MRO = Midwest Reliability Organization, RFC = Reliability First Corporation, SPP = Southwest Power Pool, WECC = Western Electricity Coordinating Council, NPCC = Northeast Power Coordinating Council, SERC = Southeast Reliability Corporation, and TRE = Texas Reliability Entity, the white space represents the intermediate region with various memberships).

Energy consumption by the stack (*E*_LAWE_ in kWh/kg H_2_) affected by voltage degradation over its lifetime was calculated using eq 1 and the amount of H_2_ production (*m*_H_2__ in kg/h) was calculated using eq 2.^33^

1 $$E_{LAWE}=\frac{\left(V+\Delta V_{\deg}\times t\right)\left(F\right)\left(2e^{-}\right)}{\left(M_{H_{2}}\right)\left(60\right)\left(60\right)}$$

2 $$m_{H_{2}}=\frac{\left(P_{RE}\right)\left(1000\right)}{E_{LAWE}}$$

where cell voltage (*V*) for the baseline stack was assumed to be 2 V and advanced stack was 1.7 V at the beginning of life.^19^ Voltage degradation rate (Δ*V*_deg_) was modeled as 3.2 millivolts/1000 h for the baseline stack and 1.4 millivolts/1000 h for the advanced stack.^19^ Lifetime (*t*) for the baseline and advanced stacks was assumed as 60,000 and 80,000 h, respectively.^34^ Among other parameters in eq 1 were Faraday’s constant (*F* = 96485.33 C/mol) and the molecular mass of H_2_ (*M*_H_2__ = 2.016 g/mol).^33^ H_2_ production correlates with power output (*P*_RE_) in megawatts, adjusted to kW.

Next, we modeled the H_2_ gas leakage during operation. A previous study estimated that H_2_ gas leakage from a LAWE on a percentage basis could be attributed to 70% from venting due to start-up and shutdown caused by wind power unavailability, 20% from H_2_ crossover, 4% from scheduled maintenance and emergency shutdown, 4% from leakages in flanges and valves of the system, and 2% from operational purging.^22^ For the three case study areas, H_2_ gas leakage (in kg/h) from venting at shutdown (*m*_H_2_,vent,shutdown_) and start-up (*m*_H_2_,vent,start‑up_) was calculated using eqs 3 and 4.^22^

3 $$m_{H_{2},vent,shutdown}=\left(V_{equip}\right)\left(\rho_{H_{2}}\right)$$

4 $$m_{H_{2},vent,start‐up}=\left(C_{H_{2},\min}\right)\left(t_{vent,start‐up}\right)$$

To reduce the possibility of the formation of an explosive gas mixture during shutdown, it was assumed that H_2_ gas was vented from the cathode-specific equipment and connected pipes. Based on data from a previous study,^22^ volume of these equipment and piping involved (*V*_equip_) was 221 m^3^ for a 100 MW LAWE, which we scaled for 48 MW LAWE in this study as 106 m^3^. Next, based on the density of H_2_ at process condition (ρ_H_2__ = 0.069 kg/m^3^) from that study, we estimated the vented amount of H_2_ gas was 7.34 kg/h. To achieve a desired purity of H_2_ (>99.8%) after starting-up, it was assumed that H_2_ gas was vented for 2 h (i.e., *t*_vent,start‑up_) while running the system at 15% capacity (i.e., *C*_H_2_,min_).^22^ H_2_ leakage due to crossover was estimated based on heat and mass balance, where 0.41 mol % H_2_ was estimated to be lost with the coproduct oxygen that typically is vented out of the system.^22^ The remaining loss of H_2_ from scheduled maintenance and emergency shutdown, leakages from flanges and valves, and operational purging were not considered in this model due to the requirement of instrument-specific measurements for their respective calculations.^22^ Finally, the estimated H_2_ gas leakages (in kg/h) were divided by the corresponding H_2_ production rates (in kg/h) obtained from eq 2 to determine the leakage rates in kg/kg H_2_.

Apart from the use of electricity by the LAWE system, the usage of other resources to produce 1 kg of H_2_ was obtained from past LCA studies (see Table S1). For example, water usage was 10–30 kg/kg H_2_ which reflects water purification, seasonal requirements for process cooling, and inefficiency of design.^12,13,19,35,36^ Liquid nitrogen for system purging during maintenance was assumed at 0.00029 kg/kg H_2_ and the electrolyte KOH was assumed at 0.00085–0.0019 kg/kg H_2_ (Table S4).^12,13,17^

#### 2.2.3 Unit Processes

We used ecoinvent 3.10 (adapted to be used in OpenLCA 2.0.4) for the inventory analysis.^37^ Among the different materials used in the stack and BoP (Figure 2b,c), US-based market unit processes were available only for steel and aluminum in ecoinvent. Therefore, to develop a US-representative inventory for the other materials, we modified existing global or rest-of-the-world market unit processes in ecoinvent by converting (1) manufacturing technologies and supply chains typically employed in the US, leveraging information from US Geological Survey’s mineral commodities inventory^38^ and US Environmental Protection Agency’s supply chain profiles,^40,41^ (2) US-based electricity, heat, and water unit processes (available in ecoinvent) used in manufacturing, and (3) US-based transport unit processes (freight, train, and shipping; also available in ecoinvent) along with corresponding distances collected from the Freight Analysis Framework Version 5 (FAF5) (Tables S2 and S3).^39^

Unlike raw materials acquisition, ecoinvent includes US-based unit processes for wind and solar electricity sources classified based on the North American Reliability Corporation (Figure 3). Therefore, for solar electricity use by the LAWE, unit process for electricity production from open-ground photovoltaic installations under the Western Electricity Coordinating Council (WECC) was used for all three case study locations. In ecoinvent, inventory for photovoltaic installation was not available for Texas Reliability Entity (TRE) although inventory for onshore wind turbine was available. As such, for wind electricity use, unit process for electricity production from >3 MW onshore wind turbines under WECC was used for Casper, Wyoming, and Daggett, California, and TRE was used for Amarillo, Texas, locations. For other resources, such as deionized water, nitrogen, and KOH, the respective global and rest of the world unit processes were modified to represent US-based electricity, heat, and water.

### 2.3 Life Cycle Impact Assessment Methods

We employed Tool for Reduction and Assessment of Chemicals and Other Environmental Impacts version 2.1 (TRACI v2.1; adapted to be used in OpenLCA 2.0.4) for the impact assessment, considering that the characterization factors under this method were developed by the US Environmental Protection Agency.^40^ The impact categories considered were: climate change (in kg CO_2_-eq/kg H_2_, based on 100-year global warming potential), acidification (in kg SO_2_-eq/kg H_2_), eutrophication (in kg N-eq/kg H_2_), ozone depletion (in kg CFC-11-eq/kg H_2_), particulate matter formation (in kg PM_2.5_-eq/kg H_2_), photochemical oxidant formation (in kg O_3_-eq/kg H_2_), freshwater ecotoxicity (in CTUe/kg H_2_), and carcinogenic human toxicity (in CTUh/kg H_2_). In order to include the climate change impact of H_2_ gas leakage as discussed in Section 2.2.2, most recent estimate of 100-year global warming potential of H_2_ of 11.6 (±2.8) kg CO_2_-eq/kg H_2_ leaked was used and added to the climate change impacts obtained from TRACI.^24^ Furthermore, to assess resource use potential (not incorporated in TRACI v2.1), we used ReCiPe 2016 v1.03, midpoint (E) (adapted to be used in OpenLCA) for the following additional impact categories: fossil energy use (in kg oil-eq/kg H_2_), land use (in m^2^·a crop/kg H_2_), materials (metals and mineral) depletion (in kg Cu-eq/kg H_2_), and water use (i.e., consumption) (in m^3^/kg H_2_).^41^

### 2.4 Interpretation

We developed the LCA model in OpenLCA 2.0.4 with 1000 Monte Carlo Simulations to incorporate the uncertainties of input materials and component data collected from the past LCA studies (Tables S4 and S5), operational model assumptions (discussed in Section 2.2.2), and the different processes within ecoinvent 3.10. Across the three life cycle stages, we compared different scenarios where baseline stack and BoP, advanced stack and BoP, and advanced stack and BoP with stack recycling utilized solar, wind, and hybrid electricity for 1 kg of H_2_ production. We interpreted these results utilizing spider plots where each of the impact categories were normalized based on a baseline scenario to identify environmental footprints and hotspots at the various life cycle stages, components levels, and materials of the LAWE. For this purpose, we followed a top-down approach, where we first compared the life cycle impacts of the 12 categories to indicate the life cycle stages with the greatest contributions across the different scenarios. Next, we compiled critical environmental impact categories by life cycle stages as well as components and materials.

Two sensitivity analyses were conducted to assess factors influencing the life cycle impacts of an LAWE system. First, an ±20% variation was applied to four key parameters—cell voltage, electricity consumption by BoP, stack lifetime, and voltage degradation rate—to identify which parameters most strongly affect the overall system performance considering that these parameters were primarily derived from best-case literature values. Specifically, literature reports cell voltages for baseline stacks in the range of 1.8–2.4 V, which aligns closely with the ±20% variation considered in this study (1.6–2.4 V).^42,43^ Second, regional sensitivity was evaluated separately for the manufacturing and O&M phases. For manufacturing, US inventories were compared with global and European data sets, capturing at a high level, variations in supply chains for materials including critical materials. In other words, the inventories are varied to reflect the sources of materials for different regions, not the assumption that the materials are sourced domestically in the US or Europe. For the O&M phase, country-level electricity production data from three leaders with significant water electrolysis deployment initiatives were compared with the US, specifically, Germany (H2Giga^44^), China (China Hydrogen Alliance^45^), and Australia (ARENA projects^46^). The approach presented is capable of capturing the influence of regional solar and wind electricity mixes and operational practices on life cycle impacts. Regional sensitivity analysis for the end-of-life phase was not conducted as all inventories including the US case leverage aggregate global and rest of the world unit processes. The end-of-life representation of key components such as steel and critical materials would be a valuable contribution in future LCA of LAWE.

## 3 Results and Discussion

### 3.1 Life Cycle Environmental Impacts of a Liquid Alkaline Water Electrolyzer

We estimated potential ranges of 12 environmental impact categories under various scenarios for the life cycle of a LAWE to produce 1 kg of H_2_ (Figure 4). The minimum and maximum values of these ranges were obtained from the 1000 Monte Carlo simulations in OpenLCA. These values are primarily driven by the type of electricity source, high magnitude of electricity used by the stacks, and its propagation to the uncertainty estimations as the ecoinvent processes for solar and wind electricity are built with log–normal distributions (Figures 4 and S1).^47–50^ For example, in the case of solar-powered LAWEs, contribution of emissions from the electricity source ranges from 91 to 94% of the total life cycle emissions of 2.57–5.09 kg of CO_2_-eq (Figure 4 and Table S6). For wind-powered LAWEs, emissions from the electricity source range from 83 to 88% of the 1.03–2.18 kg CO_2_-eq of total life cycle emissions. Further, a comparison of the different electricity sources used by the baseline stack design indicated that the solar electricity source resulted in 8 times higher impacts in water use and 15 times higher impacts in land use than that of wind electricity (Figure 5a). Remarkably, life cycle water use ranged from 0.009 to 0.16 m^3^ (2.4–42.3 gallons), mainly reflecting a best-case water consumption scenario (2.4 gallons) due to a negligible amount of water used for manufacturing wind turbines and a worst-case water consumption scenario (42.3 gallons) resulting from high water requirement in processing silicon during solar panel manufacturing (Table S6).^51^ A substantial difference was observed among scenarios for land use, ranging from 0.05 to 1.68 m^2^·a crop, reflecting a worst-case land use scenario where solar panels require considerably higher areas of land for its ground installation and a best-case land use scenario where installation of wind turbines requires significantly lower areas of land (Figure 4 and Table S6).^52^ Overall, our results align with previous LCA studies that find electricity source drives the life cycle impacts of LAWEs,^13–15,18,20^ and our analysis highlights how the energy source influences indirect land and water use impacts.

**Figure 4:**
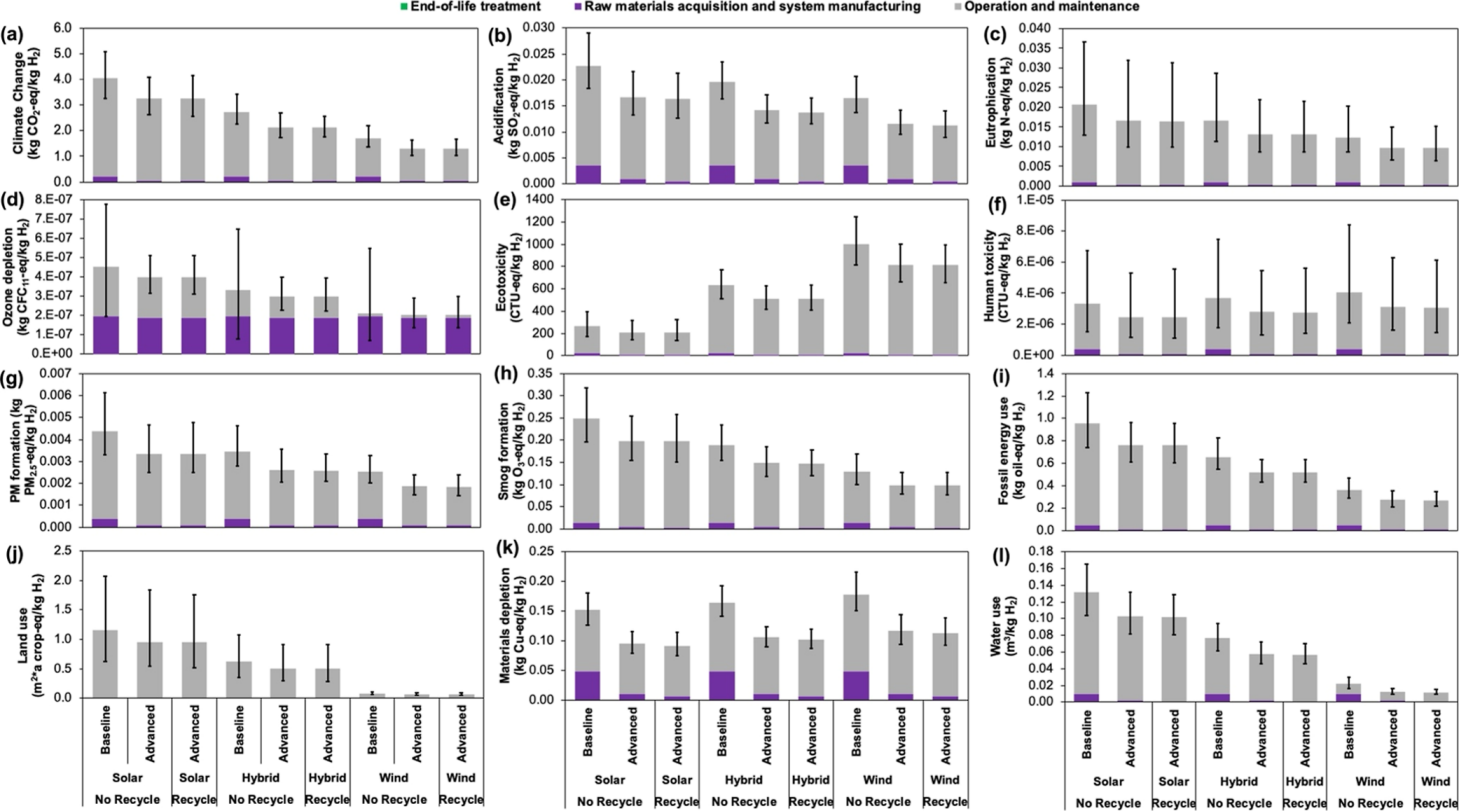
Breakdown of the 12 impact categories based on life cycle stages: (a) climate change, (b) acidification, (c) eutrophication, (d) ozone depletion potential, (e) freshwater eco-toxicity, (f) carcinogenic human toxicity, (g) particulate matter (PM) formation, (h) photochemical smog formation, (i) fossil energy use, (j) land use, (k) materials depletion, and (l) water use. Error bars represent uncertainty ranges from Monte Carlo simulations.

**Figure 5:**
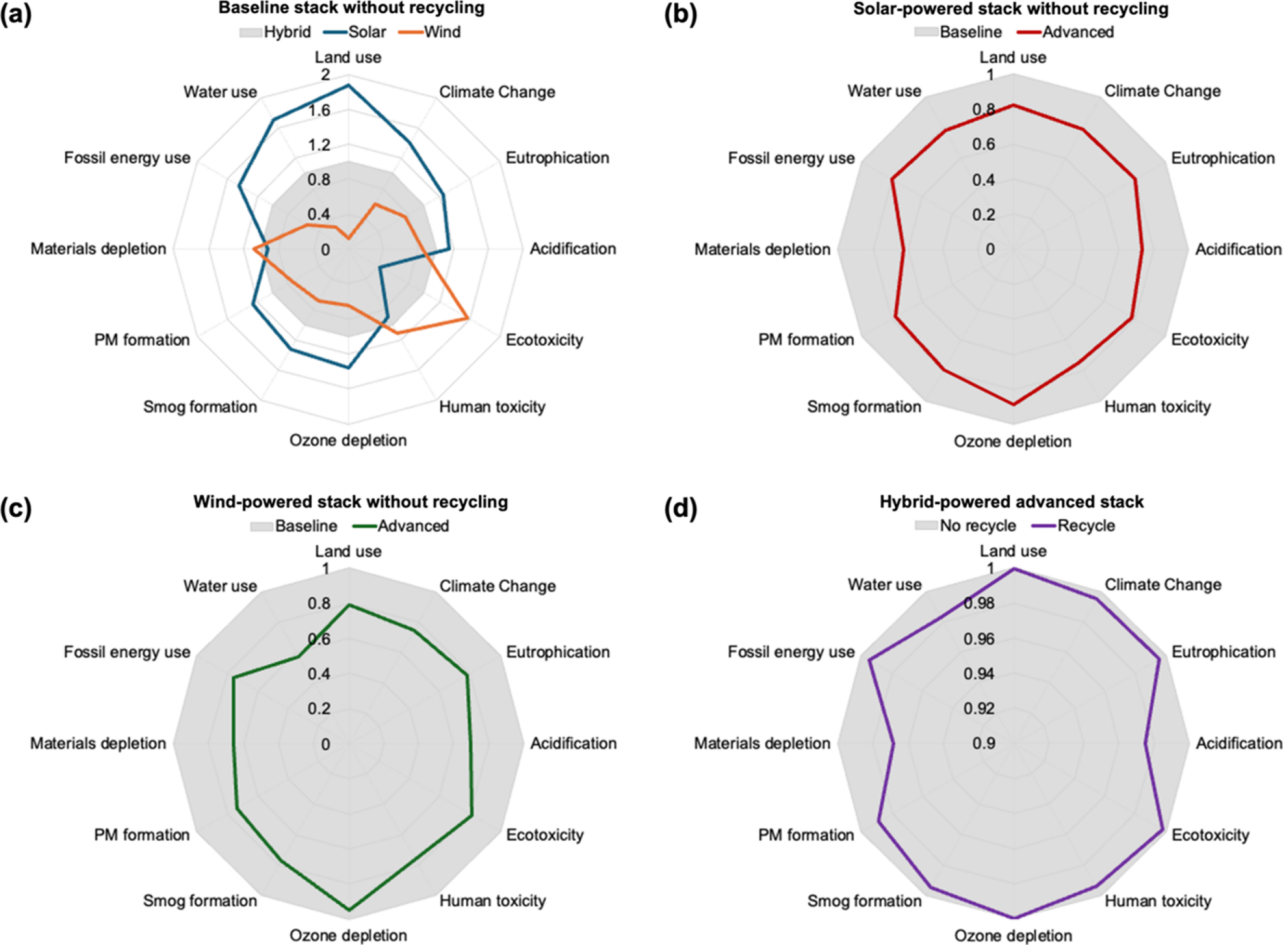
Comparison of the 12 environmental impact categories for the different scenarios: (a) baseline stack without recycling using solar versus wind versus hybrid electricity sources (benchmarked by the average impacts of using hybrid electricity), (b) solar-powered baseline versus advanced stack (benchmarked by the average impacts of baseline design), (c) wind-powered baseline versus advanced stack (benchmarked by the average impacts of baseline design), and (d) hybrid-powered advanced stack without versus with recycling (benchmarked by the average impacts of without stack recycling).

A comparison of the total life cycle impacts across the two different designs indicated that advanced stack design resulted in approximately 20–25% lower impacts for the 12 categories compared to that of the advanced stack design irrespective of the electricity source (Figure 5b,c). Such lower life cycle impacts were mainly attributed by the lower amount of electricity consumption by the advanced design (stack: 46–49 kWh/kg H_2_ and BoP: 4 kWh/kg H_2_) than that of the baseline design (stack: 54–59 kWh/kg H_2_ and BoP: 6 kWh/kg H_2_) in the O&M stage, where O&M covered more than 90% of the total impacts across the three life cycle stages (Tables S4 and S5 and Figures S2–S4). Although covering less than 10% of the total impacts across the three life cycle stages, lower embodied impacts of the advanced stack compared with the baseline stack also contributed to the lowering of the total life cycle impacts from baseline to advanced design (Figures 4 and S1). We, additionally, compared total life cycle impacts of the two different end-of-life strategies for the advanced stack design and found that incorporation of stack recycling resulted in approximately 1% lower impacts for the 12 categories, where the end-of-life impacts covered less than 1% of the total impacts across the three life cycle stages (Figures 4 and 5d). Overall, the total life cycle impacts of a LAWE for the different scenarios were primarily driven by the type of electricity for H_2_ production in the O&M stage and moderately driven by the embodied impacts of the stacks and BoP, whereas the impacts of stack recycling practices were negligible. Based on these results, we dissected the impacts from the O&M stage and the raw materials acquisition, manufacturing, and stack recycling stage below.

#### 3.1.1 Operation and Maintenance Impacts

For the O&M life cycle stage, we compared results for 12 impact categories, benchmarking by the scenario representing the baseline stack design utilizing hybrid electricity (Figure 6a). The impacts from electricity use reflect the effects of cell degradation, where the combined electricity used by the stack and BoP varied from 59 to 64 kWh/kg H_2_ for the baseline design and 49 to 52 kWh/kg H_2_ for the advanced design from the beginning to end of life (Figure S3). Electricity use not only included the effect of cell degradation exhibited through the downward trend from the beginning to end of life of the stack but also included the seasonality of the wind and solar electricity sources (Figure S2). In addition to electricity use (1.16–3.57 kg of CO_2_-eq), H_2_ gas leakage also contributed to climate change impact (0.04–0.26 kg of CO_2_-eq) (Figure 6b). Estimates of H_2_ gas leakage resulting from the variability of wind and solar energy sources indicated that the highest leakages occurred from using solar electricity (on average, 0.022 kg/kg H_2_) and lowest from using hybrid and wind electricity consumption (on average, 0.002–0.004 kg/kg H_2_) (Figures 6c and S4). These estimations are within the range of H_2_ gas leakage reported in the literature (0.002–0.034 kg/kg H_2_) that studied this phenomenon for solar- or wind-based electrolytic H_2_ production.^53–55^ H_2_ gas leakage not only results in environmental consequences, representing the second largest driver of climate change impact from LAWE (Figure 6b), but also can result in economic losses that can affect financial incentives for deploying LAWEs. Options to avoid such impacts due to H_2_ gas leakage could be grid connections and/or battery storage integration to the LAWE system.^56^

**Figure 6:**
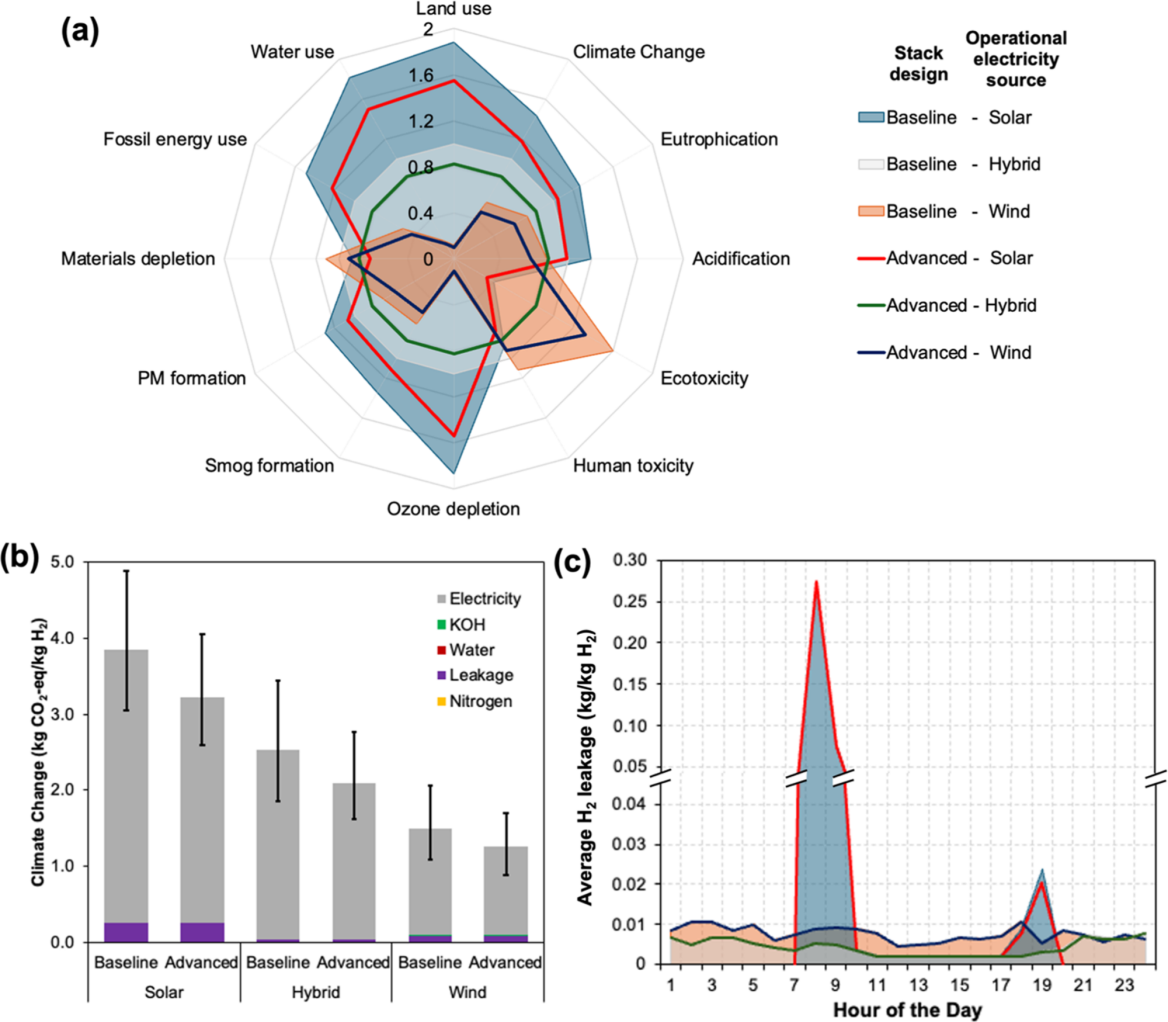
Summary of the environmental impacts for the operation and maintenance of a LAWE. (a) Normalized comparison of 12 impact categories across different scenarios, where all scenarios were normalized by the scenario representing the baseline stack with hybrid electricity. (b) Breakdown comparison of climate change impacts across different scenarios (error bars represent uncertainty ranges from Monte Carlo simulations). (c) H_2_ leakage across different scenarios.

Impact contributions from the O&M stage were substantially higher than other life cycle stages (Figure 4). However, in the case of ozone depletion, O&M stage impact was relatively lower than the embodied impacts (Figure 4). Based on the O&M stage impacts, the use of solar panels resulted in 14 times higher ozone depletion than that of the wind electricity (Figure 6a). This large difference in ozone depletion across the scenarios (1.18 × 10^–8^–3.41 × 10^–7^ kg CFC_11_-eq; Figure S1) reflected the current practices of using ozone-depleting chemicals (e.g., chlorofluorocarbons as cleaning solvents) while acquiring silicon for manufacturing solar panels.^57,58^ Among the other categories, significantly large changes in impacts across scenarios were observed for land use (0.05–2.11 m^2^·a crop), water use (0.007–0.16 m^3^), and freshwater eco-toxicity (134–1239 CTU-eq) (Figures 6a and S1). Specifically, we found that the use of solar electricity resulted in land and water use impacts 10–16 times higher than those of wind electricity (Figure 6a). These higher impacts are because of the land footprint and water consumption by manufacturing and ground installation of solar panels as compared to onshore wind turbines.^51,52^ The only category where the impact of wind electricity use was higher than that of solar electricity (4 times) was freshwater eco-toxicity (Figure 6a), which stemmed from the heavy-metal release into the water during steel production that is used in the tower construction of wind turbines unlike solar panels.^59^ The use of nitrogen, water, and KOH resulted in negligible impacts (Figures 6b and S1).

#### 3.1.2 Materials Acquisition, System Manufacturing, and Stack Recycling Impacts

For the acquisition of raw materials, manufacturing, and stack end-of-life treatment of the LAWE life cycle stage, we compared the scenarios for the 12 impact categories benchmarking by the scenario representing the baseline stack design (Figure 7a). Based on this comparison, for all impact categories except for ozone depletion, we found significant reductions (78–92%) in impacts from baseline to advanced stack design scenarios and relatively smaller reductions (4–11%) when stack recycling practices were included with the advanced stack design scenario. These reductions of impacts in the 11 categories can be predominantly attributed to the decreased use of steel and nickel in electrodes, bipolar plates, and end plates in the advanced stack design (Figure S5). Impacts from ozone depletion scarcely reduced from baseline to advanced stack design scenario, where the main contributor was tetrafluoroethylene (in plastics and rubber) used in the construction of the gasket in stacks (Figure S5). Although the estimated impacts from ozone depletion were considerably low (1.86 × 10^–7^–1.94 × 10^–7^ kg CFC_11_-eq), we would like to note that ethylene propylene diene monomer (EPDM) can alternatively be used in the construction of gaskets in the advanced stacks to lower ozone depletion.^20^ Overall, the scenario with the lowest impact across the different categories was advanced stack with recycling, where impacts in the ozone layer depletion category resulting from materials used for the gasket of the stack could potentially be improved.

**Figure 7:**
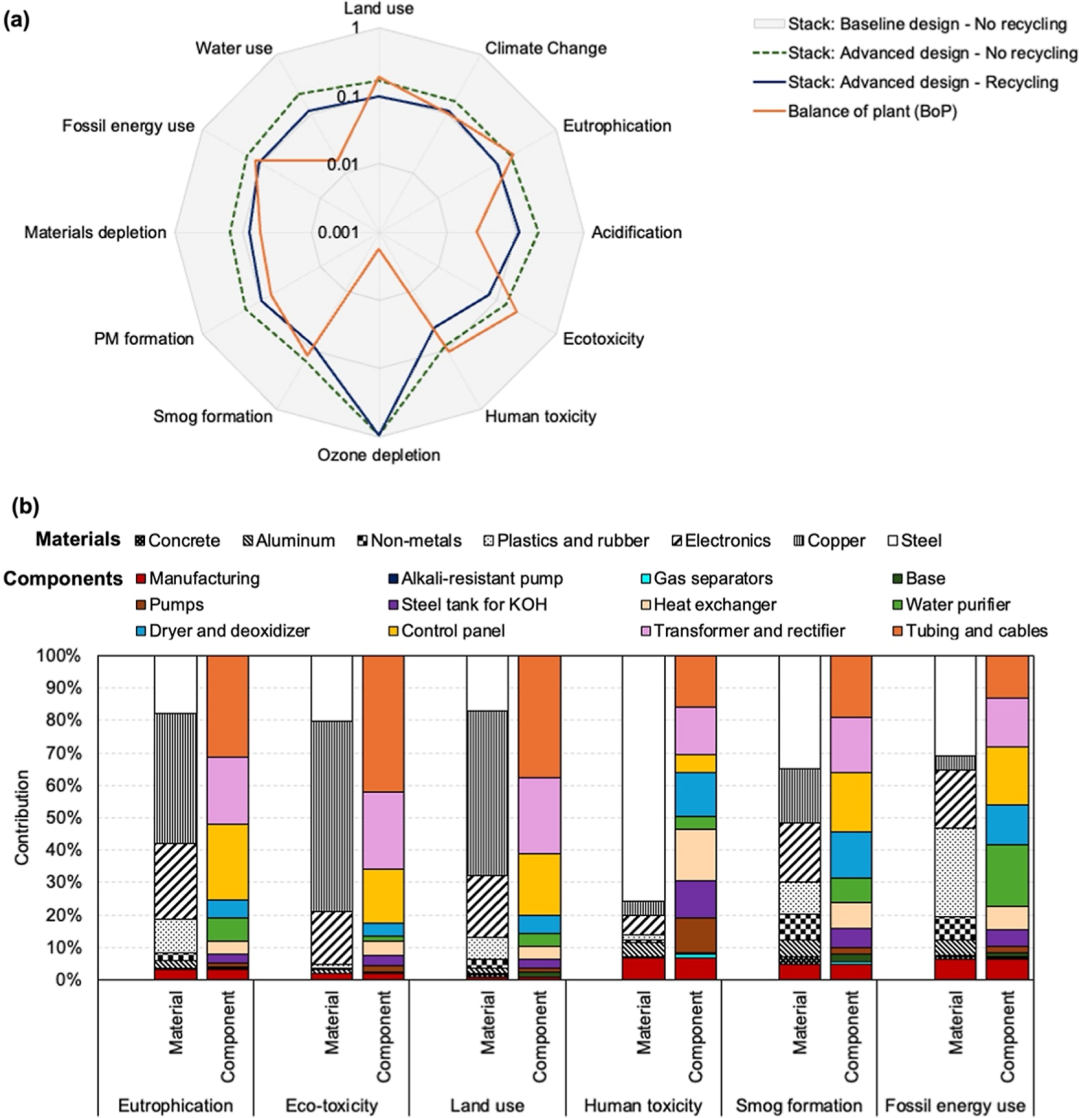
Summary of embodied environmental impacts for the acquisition of raw materials, manufacturing, and stack end-of-life treatment of a LAWE. (a) Normalized comparison of 12 impact categories across different scenarios, where all scenarios were normalized by the baseline scenario. (b) Breakdown comparisons of eutrophication, freshwater eco-toxicity, land use, carcinogenic human toxicity, smog formation, and fossil energy use contributions to BoP embodied impacts. The error bars represent the uncertainty ranges from Monte Carlo simulations.

Although the use of advanced stacks can result in significant reductions in the embodied impacts of a LAWE, the embodied impacts of BoP can be 1.5–3 times higher for eutrophication (0.0001–0.00016 vs 0.00005–0.00016 kg N-eq), freshwater eco-toxicity (2.66–5.74 vs 0.91–1.86 CTU-eq), carcinogenic human toxicity (1.85 × 10^–8^–7.43 × 10^–8^ vs 6.41 × 10^–9^–3.25 × 10^–8^ CTU-eq), smog formation (0.0012–0.0016 vs 0.0008–0.0012 kg O_3_-eq), fossil energy use (0.004–0.005 vs 0.004–0.006 kg oil-eq), and land use (0.0006–0.0011 vs 0.0003–0.0009 m^2^·a crop) (Figures 7a and S5). Across eutrophication, freshwater eco-toxicity, and land use impacts of BoP, the largest contributions came from copper (40–59%) used in the transformer and rectifier, the tubing and cables, and electronics (17–24%) used in the control panel (Figure 7b). The release of phosphate compounds and heavy metal dust from copper smelting and leaking of toxic substances in waterbodies such as lead and copper (during mining and processing) used in power electronics can be responsible for the eutrophication and freshwater eco-toxicity impacts from BoP.^60–63^ The land use impacts are related to the mining and backfilling activities for acquiring copper as well as other metals that are used in power electronics.^64^

Steel used in heat exchanger, gas separators, H_2_ dryer and deoxidizer, pumps, and tanks was the largest contributor (76%) of carcinogenic human toxicity (Figure 7b), which is caused by the use of acquisition of additives used in steel production such as ferromanganese and ferronickel.^65^ In the case of photochemical smog formation, main contributions are from steel (35%), copper (17%), and electronics (18%) (Figure 7b), potentially caused by the emissions of sulfur dioxide and nitrogen oxides during their manufacturing process, where these gases in the atmosphere react with sunlight to form ozone.^61,66–68^ Finally, the largest contributors of fossil energy use came from steel (31%), plastics and rubber (27%), and electronics (18%) (Figure 7b), all of which require high heat during manufacturing often driven by fossil fuels in conventional industries as defined in ecoinvent.^69^ Overall, these results suggested that increased attention is required to improve BoP design alongside the design of a stack for reduced embodied impacts, where potential improvements can be made to reduce the use of copper, steel, and electronics of the various BoP components.

### 3.2 Sensitivity Analysis

#### 3.2.1 Parameter Sensitivity

Sensitivity analysis with an ± 20% variation in key parameters for the best-performing scenario—an advanced stack with recycling powered by wind electricity—indicated that cell voltage had the largest influence on environmental performance, resulting in changes of up to ±18% for most impact categories and ±10% for water use (Figure 8a). This is consistent with literature reporting that operating voltage strongly affects the energy efficiency of LAWEs, highlighting that optimization of cell voltage is critical, i.e., higher voltages increase H_2_ production but also raise energy use, whereas lower voltages improve efficiency at the expense of output.^70,71^ Variations to electricity consumption by the BoP had a minor effect (<± 1.5%), while variations to stack lifetime and voltage degradation rate were largely negligible (<±1%). Ozone depletion was minimally affected for all the parameter variations (<±1%).

**Figure 8:**
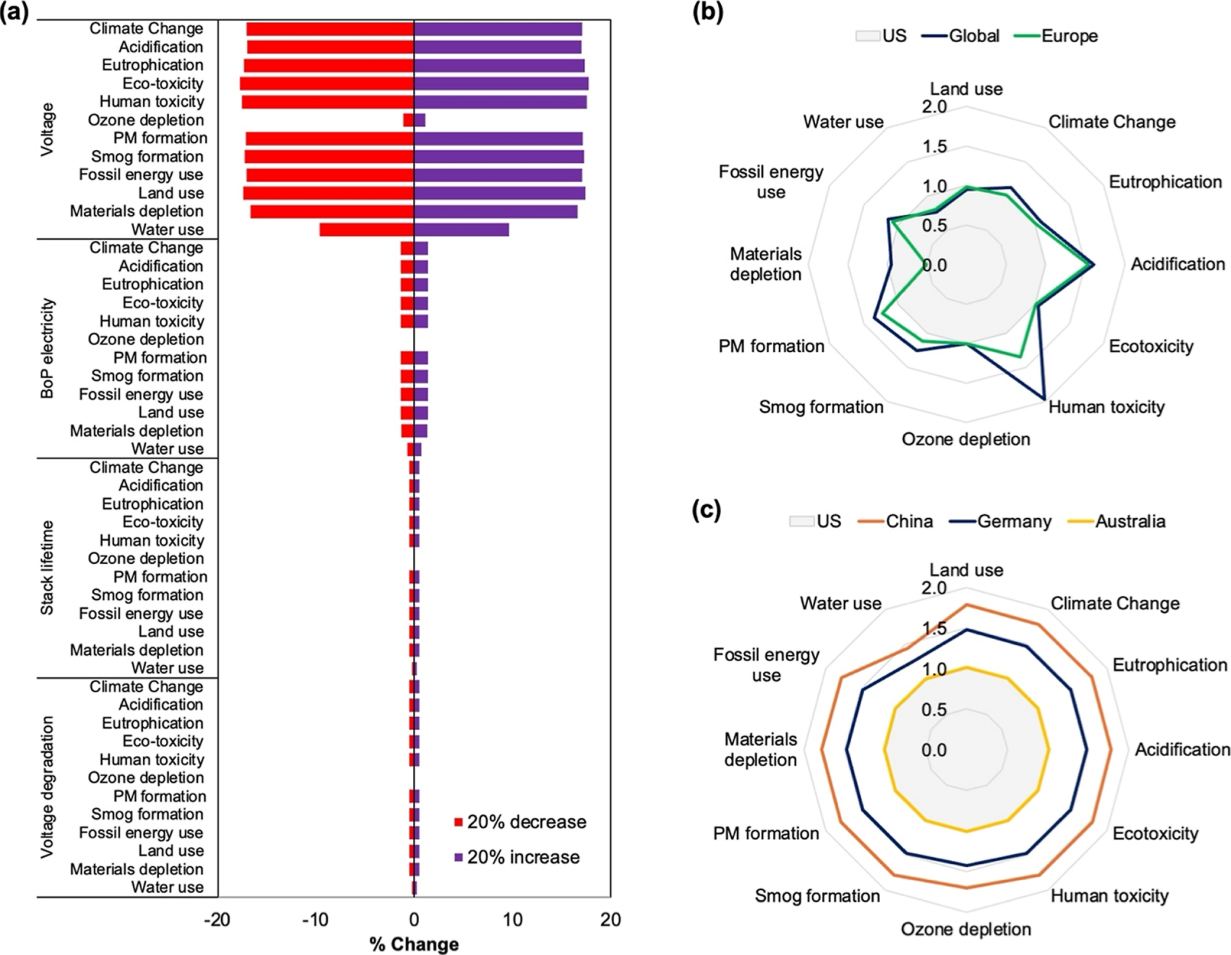
Summary of parameters and regional sensitivity analysis. (a) Tornado plot showing the results of the ±20% parameter sensitivity analysis for an advanced stack with recycling powered by a wind electricity scenario. (b) Normalized comparison of 12 impact categories for the manufacturing phase across models based on global and European unit processes versus US-representative processes. (c) Normalized comparison of 12 impact categories for the O&M phase across models based on German, Chinese, and Australian unit processes versus US-representative processes.

Sensitivity results for the solar- and hybrid-powered scenarios showed similar overall trends, with notable exceptions for ozone depletion and water use (Figure S6). For ozone depletion, a ±20% variation in cell voltage led to relatively larger changes in the hybrid (±7%) and solar (±10%) scenarios compared to the minimal changes observed in the wind scenario (Figure S6b). Similarly, for water use, the same voltage variation caused higher impacts in the hybrid (±15%) and solar (±16%) scenarios than in the wind scenario (±10%) (Figure S6l). These patterns reflect the greater environmental burdens associated with solar panel manufacturing—particularly the use of ozone-depleting chemicals for silicon production and the high water consumption—relative to wind turbines, as discussed in Section 3.1.1.

Overall, these findings highlight that precise control and optimization of cell voltage are critical for reducing energy use and environmental impacts for LAWEs, whereas uncertainties in BoP electricity, stack lifetime, and degradation rate have a limited influence.

#### 3.2.2 Regional Sensitivity

We further investigated the impacts of a LAWE for the lowest impact scenario (i.e., wind-powered advanced stack with recycling and BoP) in US, European, and Global-representative unit processes for material supply chains and for countries with clear plans for LAWE deployment (Figure 8b,c).

When considering the manufacturing and materials supply chains (Figure 8b), acidification was 60% and 55% higher, human toxicity was 50% and 35% higher, smog formation was 26% higher, and particulate matter formation was 35% and 23% higher for global and European results, respectively, compared to US inventory results. The major contributors to such differences stemmed from variations in operations of platinum group metal and nickel mining, smelting and refining, and lignite- and hard-coal-based electricity production (Figure S7). Water use was 23% and 20% higher in global and European cases than that in the US, driven primarily by the variations in hydro- and nuclear-based electricity generation (Figures 8b and S7). Material depletion was 50% lower in Europe due to reduced burdens from cobalt, nickel, copper, and iron ore mining (Figures 8b and S7).

For the O&M phase: climate change, acidification, eutrophication, human and eco-toxicity, smog and PM formation, fossil energy use, materials depletion, and land use were 48% higher with German inventories and 78% higher with Chinese inventories, while Australian inventories closely matched those of the US (Figure 8c). Key contributors included metals and clinker production, slag and ash treatment, copper and iron mining and beneficiation, electricity generation, road construction, and water production (Figure S8). Overall, these results demonstrate that both manufacturing and O&M inventories can substantially influence impact outcomes for LAWEs, with US and Australian data generally yielding the lowest environmental burdens.

These comparisons clearly indicated the importance of regionally specific modifications to inventory analysis in the raw materials acquisition and manufacturing stage of a LAWE, when focused on investigating the impacts of local manufacturing of an energy system.

### 3.3 Comparison across Hydrogen Production Pathways

Our estimate of the life cycle greenhouse emissions for the advanced LAWE design powered by solar and wind electricity is in good agreement with reported estimates for electrolysis-based H_2_ production, including LAWE, proton exchange membrane electrolyzer, and solid oxide electrolyzer technologies (Figure 9).^72^ In comparison, emissions from electrolysis technologies utilizing energy from nuclear power plants are lower by half on average, while emissions from grid-connected electrolysis are location dependent but presently much higher, sometimes on par with fossil fuel-based hydrogen production. The most common global method for H_2_ production is Steam Methane Reforming (SMR) of natural gas, with emissions slightly above 11 kg CO_2_-eq/kg H_2_. Coal gasification is another mature technology which results in even higher emissions than SMR. Carbon dioxide (CO_2_) capture and sequestration (CCS) technologies can be used to capture direct emissions, lowering life-cycle emissions to near solar-powered LAWEs depending on the end point of the captured CO_2_.^73^

**Figure 9:**
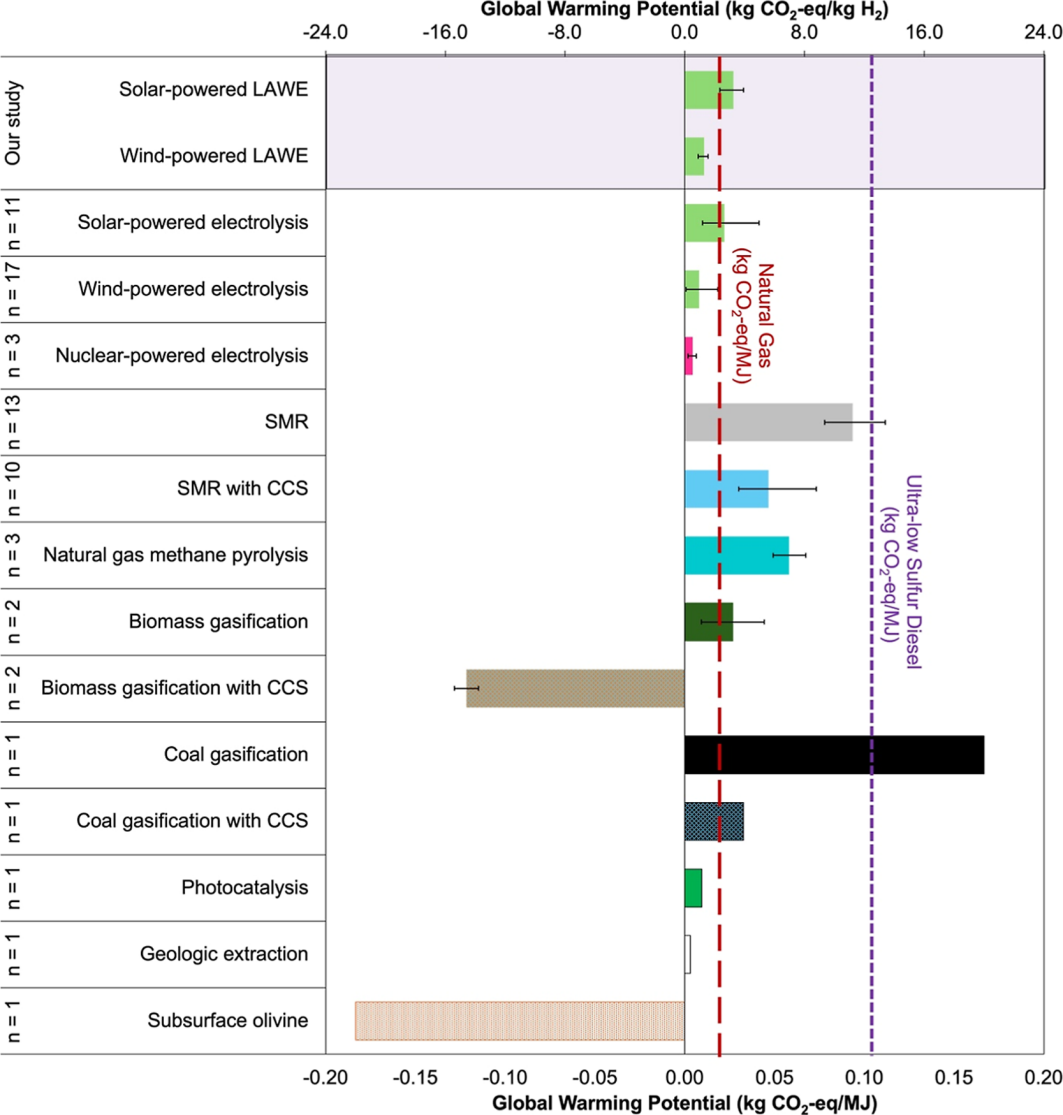
Comparison of greenhouse gas emission (global warming potential) ranges representing various H_2_ production technology pathways. SMR = natural gas Steam Methane Reforming; CCS = Carbon Capture and Storage; and *n* = sample size. Subsurface olivine serpentinization is textured to note that the value is theoretical and does not represent a life-cycle study. We use a lower heating value of hydrogen of 120.1 kg H_2_/MJ.

Other leading emerging pathways for H_2_ production include natural gas and biomethane pyrolysis, which generates H_2_ and graphite, and biomass gasification, which generates H_2_ and bio-oil, biochar, syngas, and CO_2_ when coupled with CCS. Variable feedstocks, system configurations, and LCA methodologies continue to make it challenging to set a representative value for these technologies. For example, allocation methods used in the division of emissions between the H_2_ product and byproducts can greatly affect conclusions.^74,75^ Nevertheless, pathways leveraging bioenergy offer the only method for negative carbon intensity.^76^ Other early stage options resulting in improved performance than LAWEs include photochemical water splitting, geologic H_2_ from natural reservoirs, and geologic H_2_.^77–79^ The latter results from stimulating iron-rich subsurface formations such as olivine (Fe_2_SiO_4_) through the injection of CO_2_-rich water to induce serpentinization and could have low or potentially negative overall emissions compared with electrolysis-based H_2_. Initial estimates are limited by a lack of technoeconomic analysis on such processes; however, we offer a theoretical carbon dioxide consumption of 22 kg CO_2_-eq/kg H_2_ using a simple stoichiometric equation for the serpentinization of iron-rich olivine in the presence of CO_2_-rich water.^77,78^ It is unclear what the necessary injection rate will be as well as the carbon intensity of production and injection of the water and CO_2_. We expect emissions from the removal of impurities such as methane from both types of geologic H_2_, and well construction and end of life will also affect the carbon intensity of the process.

This brief comparison highlights the promising role of LAWEs as a near-term scalable source of low-carbon H_2_ given both the low carbon intensity and the ubiquitous nature of solar and wind energy. As noted in the Introduction Section, LAWEs are not only commercially available but offer lower capital costs and operational simplicity compared with more novel electrolyzers.^4^ This allows LAWEs to serve energy, feedstock, and fuel end uses today across a range of sectors. However, lowering the carbon intensity of economic activities to the point of qualifying them as “green” products is subject to definitions that vary globally. When considering the replacement or supplementation of natural gas or diesel in end uses such as buses, trucks, or off-road vehicles and equipment, the implication for carbon emissions requires “well-to-wheel” LCA, where the efficiency of the conversion of H_2_ to operation (e.g., tonne-kilometer) is accounted for.^80,81^ For example, in trucks, wind-generated H_2_ using advanced LAWE has the potential to reduce greenhouse gas emissions by approximately 0.10 kg CO_2_-eq/tonne-km compared with diesel trucks.^82^ Using wind- or solar-powered H_2_ with advanced LAWEs in the direct reduction of iron for steel manufacturing has the potential to reduce greenhouse gas emissions by 1200–1400 kg CO_2_-eq/tonne of hot-rolled steel product compared with conventional fossil-based methods utilizing direct reduction and electric arc furnace.^82^ Given transportation and steel production contribute significantly to global emissions, even 10–20% of market adoption becomes meaningful.^83^

### 3.4 Challenges and Limitations

#### 3.4.1 Data Gaps

The LCA presented in this study relied on the best available material and component data based on scientific literature studies. Based on this available data, gaps included the allocation of materials for different components within the stack and BoP across the literature studies used for data collection (Tables S2 and S3). Importantly, these studies lacked detailed data for the materials and components of the advanced stack and BoP, unlike the baseline stack (Tables S2 and S4). Therefore, more data are needed to validate the impacts from the advanced stack design and BoP. Additionally, our LCA provided important insights into the improvement in embodied impacts of LAWEs from baseline to advanced stack design as well as differences in such impacts between advanced stack and BoP. However, further data is needed to investigate improvements in the design of BoP itself, as the embodied impacts of current BoP design resulted in more impacts than the advanced stack design in categories like eutrophication, freshwater eco-toxicity, carcinogenic human toxicity, land use, and fossil energy use (Figure 7).

Our LCA further included recycling of stacks that provided important insights into waste end-of-life treatment strategies for LAWEs and its effects on embodied impacts. Considering the negligible impacts of the end-of-life treatment processes of LAWEs, we only used global unit processes and did not modify these processes to represent US-representative processes. Additionally, due to lack of representative processes, we often used surrogate processes available in ecoinvent and indicated by the literature to build the end-of-life stage model such as the recycling processes involved for copper and nickel.

This work was conducted as part of the Department of Energy H2NEW consortium program on electrolysis innovations in 2024, which interfaces with industry partners that largely lead LAWE development. Reports emerge from stakeholder meetings bringing together water electrolysis experts including companies willing to share information on the status of technology advancements.^34^ We encourage future reviews to consider this type of non-peer-reviewed company data when verifying modeling assumptions, as we demonstrate in this analysis the limits of published data on the advanced stack and BoP, in comparison to the baseline stack (Tables S2 and S4).

#### 3.4.2 Limitations of the Study

We chose to model a 1:1 sized system (i.e., 48 MW electricity source connected to a 48 MW LAWE) due to the complexity and interdependence of full design and operational dispatch optimization for variable solar- and wind-powered LAWEs. However, LCA for different capacities needs to be investigated, considering that utilizing 1:1 sizing is unlikely to be the cost optimum for a given deployment site. Recent work highlights how optimization of solar, wind, and battery energy storage systems can vary substantially across locations at the gigawatt electrolyzer scale, with renewable capacity requirements ranging from near parity with electrolyzer capacity to more than 3-fold higher.^84^

Variables that influence economic optimization of the design and operation of behind-the-meter electrolyzers include: whether the system is coupled to the grid or fully isolated behind-the-meter and any applicable utility rate structure if grid-tied, the capital cost of the electrolyzer, batteries, and other storage as it dictates optimal capital utilization and capacity factor, performance characteristics of the electrolyzer such as peak current density and stack lifetime, hydrogen offtake constraints and requirements for the end-user, and the location of the system as it determines variable solar and wind resource quality and temporal output of wind and solar sources, influencing the oversizing ratio and storage requirements. As full system design and operational optimization are unique for each possible location and set of technology cost and performance assumptions, a full sweep of these parameters was determined to be beyond the scope of this present work. Conducting a partial or full economic optimization sweep in conjunction with LCA is a high priority for future work and will provide insights into how the trends outlined in this work apply across the multitude of different electrolyzer integration possibilities. Accordingly, our LCA represents a lower bound for operational life cycle contributions, while the accompanying Monte Carlo analysis provides insights into the variability across scenarios and strengthens confidence in the robustness of this minimum estimate. Related to this, the sensitivity analysis points to the impact of cell voltage on electricity consumption, which can be taken as an indicator of increasing life-cycle impact of wind and solar operation but does not fully capture the scope of emissions related to the proper sizing of the generation equipment.

Real-world systems are likely to use hybrid power supplies (e.g., with battery storage) and/or to be integrated with the grid. One other perspective that may also require further investigation is the impacts of electricity use and H_2_ gas leakage for grid-connected LAWEs that operate on electricity power agreements with utilities and battery storage integrations.^56^

### 3.5 Key Takeaways and Recommendations

Our LCA indicated the importance of accelerating the development of advanced stacks and further investigating the life cycle impacts of BoP within the LAWE system. The model for embodied impacts will improve as more data for advanced stack and BoP emerge with near-term deployments through the inclusion of industry and stakeholder data validation. Modifying global inventory into location-specific processes can provide more robust LCA models, and we encourage practitioners to conduct LCAs of LAWE deployments using regional inventory data gathered from suppliers.

Our LCA uniquely included an hourly model that demonstrated the effects of operational nuances of a LAWE for H_2_ production based on direct connection with variable electricity sources, such as solar, wind, and hybrid (solar and wind) electricity. Specifically, the model demonstrated not only the impacts of cell degradation of the different stacks on hourly electricity use and H_2_ gas leakage but also variabilities of the electricity source. Based on this, one important aspect that requires further investigation is the impacts of electricity supply and H_2_ gas leakage for LAWEs that utilize battery storage during the variable shutdowns and start-ups of the solar and wind electricity sources.^85,86^ Additionally, future studies should integrate economic optimization with LCA to assess how the observed trends apply across diverse electrolyzer configurations beyond the 1:1 sizing.

## Supplementary Material



## Notes

Summary of the literature review and inputs, including hourly data on stack operation, life cycle inventory, and Monte Carlo Analysis, and additional results, including life cycle assessment for 12 impact categories, sensitivity analysis, and regional analysis (PDF)

## References

[1] Sebbahi S., Assila A., Alaoui Belghiti A., Laasri S., Kaya S., Hlil E. K., Rachidi S., Hajjaji A. (2024). A Comprehensive Review
of Recent Advances in Alkaline Water Electrolysis for Hydrogen Production. Int. J. Hydrogen Energy.

[2] Dash S., K A.
S., S J., D V.
H. W., D E., Surapraraju S. K., Natarajan S. K. (2024). Advances in Green Hydrogen Production
through Alkaline Water Electrolysis: A Comprehensive Review. Int. J. Hydrogen Energy.

[3] Zhao G., Kraglund M. R., Frandsen H. L., Wulff A. C., Jensen S. H., Chen M., Graves C. R. (2020). Life Cycle
Assessment of H2O Electrolysis
Technologies. Int. J. Hydrogen Energy.

[4] Krishnan S., Koning V., Theodorus
de Groot M., de Groot A., Mendoza P. G., Junginger M., Kramer G. J. (2023). Present and Future Cost of Alkaline
and PEM Electrolyser Stacks. Int. J. Hydrogen
Energy.

[5] Phillips R., Dunnill C. W. (2016). Zero Gap Alkaline Electrolysis Cell Design for Renewable
Energy Storage as Hydrogen Gas. RSC Adv..

[6] Zeng K., Zhang D. (2010). Recent Progress in
Alkaline Water Electrolysis for Hydrogen Production
and Applications. Prog. Energy Combust. Sci..

[7] Fortune Business Insights Alkaline Water Electrolysis Market Size, Growth|Forecast [2032], FBI105436; Fortune Business Insights, 2024. https://www.fortunebusinessinsights.com/alkaline-water-electrolysis-market-105436 (accessed 2025–01–13).

[8] de
Groot M. T., Kraakman J., Garcia Barros R. L. (2022). Optimal
Operating Parameters for Advanced Alkaline Water Electrolysis. Int. J. Hydrogen Energy.

[9] Grundt T., Christiansen K. (1982). Hydrogen by
Water Electrolysis as Basis for Small Scale
Ammonia Production. A Comparison with Hydrocarbon Based Technologies. Int. J. Hydrogen Energy.

[10] Zhao P., Wang J., He W., Sun L., Li Y. (2023). Alkaline Zero
Gap Bipolar Water Electrolyzer for Hydrogen Production with Independent
Fluid Path. Energy Rep..

[11] Hellweg S., Milài Canals L. (2014). Emerging Approaches, Challenges and
Opportunities in
Life Cycle Assessment. Science.

[12] Koj J. C., Wulf C., Schreiber A., Zapp P. (2017). Site-Dependent Environmental
Impacts of Industrial Hydrogen Production by Alkaline Water Electrolysis. Energies.

[13] Wulf C., Kaltschmitt M. (2018). Hydrogen Supply
Chains for Mobility-Environmental and
Economic Assessment. Sustainability.

[14] Lee B., Cho H.-S., Kim H., Lim D., Cho W., Kim C.-H., Lim H. (2021). Integrative Techno-Economic and Environmental
Assessment for Green H2 Production by Alkaline Water Electrolysis
Based on Experimental Data. J. Environ. Chem.
Eng..

[15] Gerloff N. (2021). Comparative
Life-Cycle-Assessment Analysis of Three Major Water Electrolysis Technologies
While Applying Various Energy Scenarios for a Greener Hydrogen Production. J. Energy Storage.

[16] Lotrič A., Sekavčnik M., Kuštrin I., Mori M. (2021). Life-Cycle Assessment
of Hydrogen Technologies with the Focus on EU Critical Raw Materials
and End-of-Life Strategies. Int. J. Hydrogen
Energy.

[17] Zhang J., Wang Z., He Y., Li M., Wang X., Wang B., Zhu Y., Cen K. (2023). Comparison of Onshore/Offshore
Wind Power Hydrogen Production through Water Electrolysis by Life
Cycle Assessment. Sustain. Energy Technol. Assess..

[18] Krishnan S., Corona B., Kramer G. J., Junginger M., Koning V. (2024). Prospective LCA of Alkaline and PEM Electrolyser Systems. Int. J. Hydrogen Energy.

[19] Acevedo, Y. M. ; Prosser, J. H. ; Huya-Kouadio, J. M. ; McNamara, K. R. ; James, B. D. Hydrogen Production Cost with Alkaline Electrolysis, DOE-SA-09629-1; Strategic Analysis, Inc.: Arlington VA (United States), 2023.

[20] Iyer R. K., Prosser J. H., Kelly J. C., James B. D., Elgowainy A. (2024). Life-Cycle
Analysis of Hydrogen Production from Water Electrolyzers. Int. J. Hydrogen Energy.

[21] Lim D., Lee B., Lee H., Byun M., Cho H.-S., Cho W., Kim C.-H., Brigljević B., Lim H. (2021). Impact of Voltage Degradation
in Water Electrolyzers on Sustainability of Synthetic Natural Gas
Production: Energy, Economic, and Environmental Analysis. Energy Convers. Manage..

[22] Kaur, A. Hydrogen Emissions from an Electrolysis Unit. In Master of Science in Sustainable Energy Technology; Delft University of Technology: Delft, Netherlands, 2023. https://repository.tudelft.nl/file/File_209db7cf-1093-47bd-b030-debbb96f0290?preview=1.

[23] Ocko I. B., Hamburg S. P. (2022). Climate Consequences
of Hydrogen Emissions. Atmos. Chem. Phys..

[24] Sand M., Skeie R. B., Sandstad M., Krishnan S., Myhre G., Bryant H., Derwent R., Hauglustaine D., Paulot F., Prather M., Stevenson D. (2023). A Multi-Model
Assessment of the Global Warming Potential of Hydrogen. Commun. Earth Environ..

[25] US DOE . Funding Selections for Clean Hydrogen Electrolysis, Manufacturing, and Recycling Activities under the Bipartisan Infrastructure Law; Office of Energy Efficiency and Renewable Energy. https://www.energy.gov/eere/fuelcells/bipartisan-infrastructure-law-clean-hydrogen-electrolysis-manufacturing-and-0 (accessed 2024–09–16.

[26] US Department of Treasury . U.S. Department of the Treasury Releases Final Rules for Clean Hydrogen Production Tax Credit; U.S. Department of the Treasury. https://home.treasury.gov/news/press-releases/jy2768 (accessed 2025–01–13.

[27] International Organization for Standardization . ISO 14044:2006, Environmental Management—Life Cycle Assessment—Requirements and Guidelines, 2006. https://www.iso.org/standard/38498.html (accessed 2024–09–24).

[28] Curran, M. A. Overview of Goal and Scope Definition in Life Cycle Assessment. In Goal and Scope Definition in Life Cycle Assessment; Curran, M. A. , Ed.; Springer Netherlands: Dordrecht, 2017; pp 1–62.

[29] Razmjooei F., Liu T., Azevedo D. A., Hadjixenophontos E., Reissner R., Schiller G., Ansar S. A., Friedrich K. A. (2020). Improving Plasma Sprayed Raney-Type
Nickel-Molybdenum Electrodes towards High-Performance Hydrogen Evolution
in Alkaline Medium. Sci. Rep..

[30] USGS . Minerals Yearbook—Metals and Minerals; U.S. Geological Survey. https://www.usgs.gov/centers/national-minerals-information-center/minerals-yearbook-metals-and-minerals#S (accessed 2024–10–14.

[31] US EPA . Plastics: Material-Specific Data; United States Environmental Protection Agency (US EPA). https://www.epa.gov/facts-and-figures-about-materials-waste-and-recycling/plastics-material-specific-data (accessed 2024–10–14.

[32] NREL . Pysam, 2024. http://github.com/nrel/pysam.

[33] Lee H., Gu J., Lee B., Cho H.-S., Lim H. (2023). Prognostics and Health
Management of Alkaline Water Electrolyzer: Techno-Economic Analysis
Considering Replacement Moment. Energy and AI.

[34] US DOE . Advanced Liquid Alkaline Water Electrolysis Experts Meeting; Office of Energy Efficiency & Renewable Energy, US Department of Energy: Washington, D.C., 2022. https://www.energy.gov/sites/default/files/2022-04/liquid-alkaline-electrolysis-experts-meeting-report.pdf (accessed 2024–10–29).

[35] Kaitlyn, R. ; Tessa, W. ; Thomas, K. ; Chathurika, G. Hydrogen Reality Check: Distilling Green Hydrogen’s Water Consumption; Rocky Mountain Institute: Denver, CO, 2023. https://rmi.org/hydrogen-reality-check-distilling-green-hydrogens-water-consumption/ (accessed 2024–10–10).

[36] Valdez, T. Electrolyzers & Water: Powering the World with Green Hydrogen—Plug Power; Plug Power. https://www.plugpower.com/water-electrolysis-powering-the-world-with-green-hydrogen/ (accessed 2024–11–05.

[37] Wernet G., Bauer C., Steubing B., Reinhard J., Moreno-Ruiz E., Weidema B. (2016). The Ecoinvent Database Version 3 (Part I): Overview
and Methodology. Int. J. Life Cycle Assess..

[38] USGS . Mineral Industry Surveys|Industry Data; United States Geological Survey (USGS). https://www.usgs.gov/centers/national-minerals-information-center/mineral-industry-surveys (accessed 2025–02–14.

[39] Hwang, H.-L. ; Lim, H. ; Chin, S.-M. ; Uddin, M. ; Biehl, A. ; Xie, F. ; Hargrove, S. ; Liu, Y. ; Wang, R. Freight Analysis Framework Version 5 (FAF5) Base Year 2017 Data Development Technical Report, ORNL/TM-2021/2154; Oak Ridge National Laboratory (ORNL): Oak Ridge, TN (United States), 2021.

[40] Bare, J. TRACI 2.1: Tool for the Reduction and Assessment of Chemical and Other Environmental Impacts Version 2.1, User Manual EPA/600/R-12/554; United States Environmental Protection Agency: Cincinnati, OH, 2012. https://nepis.epa.gov/Adobe/PDF/P100HN53.pdf (accessed 2022–08–07.

[41] Dekker E., Zijp M. C., van de Kamp M. E., Temme E. H. M., van
Zelm R. (2020). A Taste of the New ReCiPe for Life Cycle Assessment: Consequences
of the Updated Impact Assessment Method on Food Product LCAs. Int. J. Life Cycle Assess..

[42] Xiang C., Papadantonakis K. M., Lewis N. S. (2016). Principles and Implementations
of
Electrolysis Systems for Water Splitting. Mater.
Horiz..

[43] El-Shafie M. (2023). Hydrogen Production
by Water Electrolysis Technologies: A Review. Results Eng..

[44] Büsch L., Jakschik M., Syniawa D., Masuhr C., Christ L., Schachtsiek J., Haalck K., Nerlich L., Frömsdorf E., Schirmack N., Ebert B., Kirty C., Adler P., Schüppstuhl T., Kuhlenkötter B. (2024). HyPLANT100: Industrialization from
Assembly to the Construction Site for Gigawatt Electrolysis. Hydrogen.

[45] Huang Y., Zhou Y., Zhong R., Wei C., Zhu B. (2024). Hydrogen Energy
Development in China: Potential Assessment and Policy Implications. Int. J. Hydrogen Energy.

[46] CSIRO . Hydrogen Electrolyser Manufacturing Report; Commonwealth Scientific and Industrial Research Organisation (CSIRO): Canberra, Australia, 2024. https://www.csiro.au/en/work-with-us/services/consultancy-strategic-advice-services/CSIRO-futures/Energy/Hydrogen-Electrolyser-Manufacturing-Report (accessed 2025–09–18.

[47] Burkhardt J. J., Heath G., Cohen E. (2012). Life Cycle Greenhouse
Gas Emissions of Trough and Tower Concentrating Solar Power Electricity
Generation. J. Ind. Ecol..

[48] Nugent D., Sovacool B. K. (2014). Assessing the Lifecycle
Greenhouse Gas Emissions from
Solar PV and Wind Energy: A Critical Meta-Survey. Energy Policy.

[49] Ciroth A., Fleischer G., Steinbach J. (2004). Uncertainty Calculation in Life Cycle
Assessments. Int. J. Life Cycle Assess..

[50] Weidema, B. P. ; Bauer, C. ; Hischier, R. ; Mutel, C. L. ; Nemecek, T. ; Reinhard, J. ; Vadenbo, C. O. ; Wernet, G. Overview and Methodology: Data Quality Guidance for the Ecoinvent Database Version 3; Ecoinvent Report Vol. 3 No. 1; Swiss Centre for Life Cycle Inventories: St. Gallen, Denmark, 2013. https://vbn.aau.dk/ws/files/176769045/overview_and_methodology.pdf.

[51] Meldrum J., Nettles-Anderson S., Heath G., Macknick J. (2013). Life Cycle Water Use
for Electricity Generation: A Review and Harmonization of Literature
Estimates. Environ. Res. Lett..

[52] Ritchie, H. How Does the Land Use of Different Electricity Sources Compare?; Our World in Data, 2022.

[53] Frazer-Nash . Fugitive Hydrogen Emissions in a Future Hydrogen Economy; FNC 012865-53172R Issue 1; Frazer-Nash Consultancy: United Kingdom, 2022. https://assets.publishing.service.gov.uk/media/624ec79cd3bf7f600d4055d1/fugitive-hydrogen-emissions-future-hydrogen-economy.pdf.

[54] Arrigoni, A. ; Bravo, D. L. Hydrogen Emissions from a Hydrogen Economy and Their Potential Global Warming Impact; JRC; Publications Office of the European Union: Luxembourg, 2022.

[55] Harrison, K. ; Peters, M. Renewable Electrolysis Integrated System Development & Testing; NREL, 2013. https://www.hydrogen.energy.gov/docs/hydrogenprogramlibraries/pdfs/review13/pd031_harrison_2013_o.pdf (accessed 2025–02–21.

[56] Chaney, C. ; Pastoria, C. To Connect or Not to Connect: Demystifying Hydrogen Power Procurement Options, Risks, and Opportunities; Rocky Mountain Institute (RMI). https://rmi.org/to-connect-or-not-to-connect-demystifying-hydrogen-power-procurement-options-risks-and-opportunities/(accessed 2025–02–21.

[57] Torres J. F., Petrakopoulou F. (2022). A Closer Look at the Environmental Impact of Solar
and Wind Energy. Glob Chall.

[58] Mahmud M. A. P., Farjana S. H. (2022). Comparative Life Cycle Environmental Impact Assessment
of Renewable Electricity Generation Systems: A Practical Approach
towards Europe, North America and Oceania. Renewable
Energy.

[59] Greening B., Azapagic A. (2013). Environmental Impacts
of Micro-Wind Turbines and Their
Potential to Contribute to UK Climate Change Targets. Energy.

[60] ICA . Copper Environmental Profile; International Copper Alliance, 2023. https://internationalcopper.org/wp-content/uploads/2023/05/ICA-LCI-GlobalSummary-202305-F.pdf (accessed 2025–02–19.

[61] Izydorczyk G., Mikula K., Skrzypczak D., Moustakas K., Witek-Krowiak A., Chojnacka K. (2021). Potential Environmental Pollution
from Copper Metallurgy and Methods of Management. Environ. Res..

[62] Hong J., Shi W., Wang Y., Chen W., Li X. (2015). Life Cycle Assessment
of Electronic Waste Treatment. Waste Manage..

[63] Brunhoferová H., Trecáková T., Kočí V. (2025). Life Cycle
Assessment of Electronic, Electric and Nonelectric Detonators; a Site-Specific
Case for Czech Republic. Heliyon.

[64] Tao M., Nie K., Zhao R., Shi Y., Cao W. (2022). Environmental Impact
of Mining and Beneficiation of Copper Sulphate Mine Based on Life
Cycle Assessment. Environ. Sci. Pollut. Res..

[65] Harasymchuk I., Kočí V., Trecáková T. (2024). Research of the Life
Cycle for Two Most Common Routes of Steel Production with a Focus
on the Impact to the Human Body. Int. J. Sustain.
Eng..

[66] US EPA . Overview of the Human Health and Environmental Effects of Power Generation: Focus on Sulfur Dioxide, Nitrogen Oxides, and Mercury; United States Environmental Protection Agency (US EPA), 2002. https://archive.epa.gov/clearskies/web/pdf/overview.pdf (accessed 2025–02–18.

[67] US EPA . 2011–2017 Greenhouse Gas Reporting Program Profile: Electronics Manufacturing Sector; United States Environmental Protection Agency (US EPA), 2018. https://www.epa.gov/sites/default/files/2018-10/documents/electronics_manufacturing_2017_industrial_profile.pdf (accessed 2025–02–18.

[68] Tang L., Xue X., Jia M., Jing H., Wang T., Zhen R., Huang M., Tian J., Guo J., Li L., Bo X., Wang S. (2020). Iron and Steel Industry Emissions and Contribution
to the Air Quality in China. Atmos. Environ..

[69] Friedemann, A. J. Manufacturing Uses Over Half of All Fossil Energy. In Life after Fossil Fuels: A Reality Check on Alternative Energy; Friedemann, A. J. , Ed.; Springer International Publishing: Cham, 2021; pp 51–63.

[70] Xia Y., Cheng H., He H., Wei W. (2023). Efficiency and Consistency
Enhancement for Alkaline Electrolyzers Driven by Renewable Energy
Sources. Commun. Eng..

[71] Benghanem M., Almohamadi H., Haddad S., Mellit A., Chettibi N. (2024). The Effect
of Voltage and Electrode Types on Hydrogen Production Powered by Photovoltaic
System Using Alkaline and PEM Electrolyzers. Int. J. Hydrogen Energy.

[72] Maniscalco M. P., Longo S., Cellura M., Miccichè G., Ferraro M. (2024). Critical Review of Life Cycle Assessment
of Hydrogen
Production Pathways. Environments.

[73] AlHumaidan F. S., Absi Halabi M., Rana M. S., Vinoba M. (2023). Blue Hydrogen:
Current
Status and Future Technologies. Energy Convers.
Manage..

[74] Pangestu M. R. G., Malaibari Z., Muhammad A., Al-Rowaili F. N., Zahid U. (2024). Comprehensive Review
on Methane Pyrolysis for Sustainable Hydrogen
Production. Energy Fuels.

[75] Susmozas A., Iribarren D., Zapp P., Linβen J., Dufour J. (2016). Life-Cycle Performance
of Hydrogen Production via Indirect
Biomass Gasification with CO2 Capture. Int.
J. Hydrogen Energy.

[76] Nordahl S. L., Devkota J. P., Amirebrahimi J., Smith S. J., Breunig H. M., Preble C. V., Satchwell A. J., Jin L., Brown N. J., Kirchstetter T. W., Scown C. D. (2020). Life-Cycle Greenhouse
Gas Emissions
and Human Health Trade-Offs of Organic Waste Management Strategies. Environ. Sci. Technol..

[77] Brandt A. R. (2023). Greenhouse
Gas Intensity of Natural Hydrogen Produced from Subsurface Geologic
Accumulations. Joule.

[78] Yedinak E. M. (2022). The Curious
Case of Geologic Hydrogen: Assessing Its Potential as a Near-Term
Clean Energy Source. Joule.

[79] Collins S., Acevedo Y., Esposito D. V., Bala Chandran R., Ardo S., James B., Breunig H. (2025). Levelized
cost and
carbon intensity of solar hydrogen production *via* water splitting using a scalable and intrinsically safe photocatalytic
Z-scheme raceway system. Energy Environ. Sci..

[80] Littlefield, J. ; Augustine, D. ; Pegallapati, A. ; Zaimes, G. ; Rai, S. ; Cooney, G. ; Skone, T. Life Cycle Analysis of Natural Gas Extraction and Power Generation, DOE/NETL-2019/2039; National Energy Technology Laboratory (NETL): Pittsburgh, PA, USA, 2019. https://www.netl.doe.gov/energy-analysis/details (accessed 2025–06–02.

[81] CARB . CA-GREET3.0 Lookup Table Pathways—Technical Support Documentation; California Air Resources Board (CARB): Sacramento, CA, USA, 2023. https://ww2.arb.ca.gov/sites/default/files/classic/fuels/lcfs/ca-greet/lut_update_2023_2.pdf?utm_source=chatgpt.com (accessed 2025–06–02.

[82] Goita E. G., Beagle E. A., Nasta A. N., Wissmiller D. L., Ravikumar A., Webber M. E. (2025). Effect of Hydrogen Leakage on the
Life Cycle Climate Impacts of Hydrogen Supply Chains. Commun. Earth Environ..

[83] Elgowainy, A. ; Mintz, M. ; Lee, U. ; Stephens, T. ; Sun, P. ; Reddi, K. ; Zhou, Y. ; Zang, G. ; Ruth, M. ; Jadun, P. ; Connelly, E. ; Boardman, R. Assessment of Potential Future Demands for Hydrogen in the United States, ANL-20/35; Argonne National Lab. (ANL): Argonne, IL (United States), 2020.

[84] Reznicek E. P., Koleva M. N., King J., Kotarbinski M., Grant E., Vijayshankar S., Brunik K., Thomas J., Gupta A., Hammond S., Singh V., Tusing R., Sun P., Lee K., Elgowainy A., Breunig H., Rosner F., Pinto J. O. P. (2025). Techno-Economic Analysis of Low-Carbon Hydrogen Production
Pathways for Decarbonizing Steel and Ammonia Production. Cell Rep. Sust..

[85] Yan Z., Hitt J. L., Turner J. A., Mallouk T. E. (2020). Renewable Electricity
Storage Using Electrolysis. Proc. Natl. Acad.
Sci. U.S.A..

[86] Chamout M. W., Perl A., Hengeveld E. J., Aué J. j. (2024). Simulation
and Analysis of Hybrid Hydrogen-Battery Renewable Energy Storage for
off-Electric-Grid Dutch Household System. Int.
J. Hydrogen Energy.

