# Supplementary material for: Liquid Alkaline Water Electrolyzers: Comparing Performance across Design, Operation, and End-of-Life Scenarios
Source: Environ Sci Technol. 2025 Oct 10;59(41):21941–56. doi: 10.1021/acs.est.5c07500 (PMC12550807; doi:10.1021/acs.est.5c07500)
Supplement: Supplementary file 1 [file es5c07500_si_001.pdf]

Liquid Alkaline Water Electrolyzers: Comparing Performance Across Design, Operation, and End-of-Life Scenarios

Mohammed T. Zaki[1], Smith, Colby.[2], Badgett, Alex.[2], and Hanna M. Breunig[1,*]

1. Energy Analysis and Environmental Impacts Division, Energy Technology Area, Lawrence Berkeley National Laboratory, Berkeley, California, 94720, U.S.A.
2. Strategic Energy Analysis Center, National Renewable Energy Laboratory, Golden, Colorado, 80401, U.S.A.

*Corresponding Author: Hanna M. Breunig (hannabreunig@lbl.gov)

Supplemental Information

List of Contents:

Section S1 Literature review of LAWE LCA studies.
Table S1 A summary of LAWE LCA studies in literature.
Table S2 Literature-based material weights for manufacturing baseline and advanced stacks along with their end-of-life treatment.
Table S3 Literature-based material weights for manufacturing balance of plant along with their end-of-life treatment.
Table S4 Material weights used for Monte Carlo simulation of manufacturing baseline and advanced stacks.
Table S5 Material weights used for Monte Carlo simulation of manufacturing balance of plant.
Table S6 Overall life cycle environmental impacts to produce 1 kg H_2 across various scenarios.
Figure S1 Breakdown of the 12 impact categories based on the different contributors of the operation and maintenance stage of the life cycle.
Figure S2 Hourly stack energy use over their lifetimes.
Figure S3 Hourly hydrogen production over stack lifetimes.
Figure S4 Hourly hydrogen leakage over stack lifetimes.
Figure S5 Breakdown of the 12 embodied impact categories based on the different components and materials of stacks and balance of plant.
Figure S6 Sensitivity analysis of overall life cycle results for the 12 impact categories and different scenarios.
Figure S7 Contribution analysis of manufacturing advanced stack with recycling using US-representative, global, and European inventories.
Figure S8 Contribution analysis of O&M of advanced using US-representative, German, Chinese, and Australian inventories.

Section S1 Literature review of LAWE LCA studies.

We conducted a literature search and identified 10 LCA studies of LAWE from 2017-2024 that reported inventory data at various level of details (Table S1). Within the raw materials acquisition and manufacturing stage of the LAWE life cycle, the reviewed studies mainly focused on quantifying the environmental impacts of the stack, where limited studies evaluated BoP and manufacturing of the stack and BoP (Table S1). Although most of these studies utilized Ecoinvent to define the raw materials acquisition, these processes were based on either European or Global market. This is understandable considering that Ecoinvent although includes a comprehensive database for inventory analysis, in many cases it lacks US representative market processes. The US-based study utilized GREET, which only provided greenhouse gas emissions and not non-carbon emissions like acidification, eutrophication or impacts on available resources like fossil fuel, land, and water.[1] In the O&M phase, studies assessed the impact of electricity use on current and future grid mainly in European regions for electrolytic H_2 production (Table S1). In addition to electricity use, the use of other resources included deionized water for electrolysis, potassium hydroxide (KOH) as the electrolyte, nitrogen for electrolyzer purging, and steam for heating up the electrolyzer during ramp up of the system (Table S1).[2] However, aspects of LAWE O&M that might be critical for long-term environmental sustainability have not been considered in these LCA studies. For example, voltage degradation can result in increased electricity consumption, thereby resulting in higher emissions.[3] Additionally, H_2 crossover during regular operation and venting during shutdowns can result in H_2 leakage, which has recently come to attention due its potential to cause global warming.[4–6] The end-of-life treatment phase of LAWE waste materials (after decommissioning) has been studied the least where only one study evaluated the disposal and recycling of LAWE waste materials in Europe (Table S1).

Table S1 A summary of LAWE LCA studies in literature (*Stack and BoP components are illustrated in Figure 2a; BoP = Balance of Plant, N_2 = Nitrogen, and KOH = Potassium Hydroxide).

Life cycle phases							Source
**Raw materials acquisition and equipment manufacturing (cradle)**			**Operation and maintenance (gate)**			**End-of-life treatment (grave)**	
Stack design	*Material unit weight data availability**		*Electricity data*		*Other resources usage data*	*Process data availability*	
	Stack	*BoP*	*Source*	*Use*			
Baseline	Component-specific materials	None	Average German, Austrian, and Spanish grid mix during 2015 - 2035	Average unit consumption	Average unit consumption of water, N_2, and KOH	None	[2]
Baseline	Materials without specific components	None	German wind power guaranteed by green certificate for 2032	Average unit consumption	Average unit consumption of water and KOH	None	[7]
Baseline	Component-specific materials	None	Average South Korean grid mix for 2020, 2030, 2050, and full renewable	Process model-based Unit consumption	Average unit consumption of water, N_2, and KOH	None	[8]
Baseline	Materials without specific components and stack manufacturing	Component-specific materials and BoP manufacturing	Average German grid mix for 2020, 2030, 2050, and full renewable	Average unit consumption	Average unit consumption of water and KOH	Material-specific disposal	[9]
Baseline	Component-specific materials	None	None	None	None	Material-specific disposal	[10]
Baseline	Materials without specific components	None	Chinese onshore wind power in Inner Mongolia and offshore wind power in Xinghua Bay	Average unit consumption	Average unit consumption of water and KOH	None	[11]
Baseline and advanced	Component-specific materials	Materials without specific components	Average Dutch grid mix and wind power in North Sea during 2020 and 2030	Average unit consumption	Average unit consumption of water	None	[12]
Baseline and advanced	Component-specific materials and stack manufacturing	None	Average United States grid mix, wind, solar, and nuclear during 2021	Process model-based Unit consumption	Water	None	[1,13]

Table S2 Literature-based material weights for manufacturing baseline and advanced stacks along with their transportation and end-of-life treatment.

Sub-component	Materials	Weights (kg/kg H_2)								Transport (ton-miles)			End-of-life treatment (%)		
		Baseline design						*Advanced design*		*Rail*	*Truck*	*Sea*	*Recycle*	*Landfill*	*Incineration*
		[2,8]	[7]	[9]	[11]	[12]	[1,13]	[12]	[1,13]	[14]			[10,15,16]		
Cathode, Anode, and Frame	Steel	2.04E-02	6.62E-03	1.96E-02	1.84E-02	2.16E-02				ecoinvent US default			44	56	0
	Nickel	1.94E-03	1.03E-04	2.80E-03	2.01E-03	1.26E-03	1.93E-03	7.01E-04	5.49E-04	0.57	0.28	0.84	49	51	0
	Aluminum	4.59E-05	3.23E-05		6.02E-05			2.05E-05		ecoinvent US default			56	44	0
	Copper	2.04E-04			2.68E-04				7.35E-06	0.33	0.33	0	68	32	0
	Glass fiber reinforced plastic							1.11E-04	6.93E-05	0.42	0.12	0	0	100	0
	Polypropylene						2.54E-05		7.00E-06	0.72	0.25	0	9	76	15
Diaphragm, Zirfon	Zirconium oxide	1.12E-04		7.10E-05	1.20E-04	1.43E-04		1.29E-05		0.72	0.25	0	0	100	0
	Polyphenylene sulfide	3.47E-05			4.02E-05	4.76E-05		4.28E-06		0.72	0.25	0	9	76	15
	Polysulfone	2.65E-05		4.74E-05		3.17E-05		2.86E-06		0.72	0.25	0	9	76	15
Gasket	Polyethylene	7.96E-05	2.56E-05							0.72	0.25	0	9	76	15
	Tetrafluoroethylene	7.96E-06		1.40E-04	1.04E-05	8.37E-05		5.77E-05		0.72	0.25	0	0	100	0
	Graphite	4.39E-05			5.76E-05					0.72	0.25	0	0	100	0
	Acrylonitrile-butadiene-styrene copolymer	1.63E-05			2.14E-05					0.72	0.25	0	0	0	100
	Ethylene propylene diene terpolymer								3.35E-05	0.72	0.25	0	0	100	0
Bipolar plate	Steel					4.00E-02		4.13E-03		ecoinvent US default			44	56	0
	Nickel					1.47E-03	1.46E-03	1.68E-04	1.32E-04	0.57	0.28	0.84	49	51	0
End plate	Steel					5.21E-03	4.77E-04	6.60E-04	2.30E-04	ecoinvent US default			44	56	0

Note: Overall energy required for the manufacturing of the baseline stack was 1.94E-02 kWh/kg H_2 and advanced stack was 7.70E-03 kWh/kg H_2.[1,13]

Table S3 Literature-based material weights for manufacturing balance of plant along with their transportation and end-of-life treatment.

**Sub-component**	**Materials**	**Weights (kg/kg H_2)**				**Transport (ton-miles)**			**End-of-life treatment (%)**		
		Baseline design			*Advanced design*	*Rail*	*Truck*	*Sea*	*Recycle*	*Landfill*	*Incineration*
		[9]	[11]	[12]	[12]	[14]			[10,15,16]		
Water purifier and feed tank	Steel	2.26E-04				ecoinvent US default			56	44	0
	Polyethylene	4.52E-04				0.72	0.25	0	9	76	15
Control panel	Electronics	9.72E-05		3.34E-04	3.30E-04	0.25	0.45	0	0	0	100
Transformer and rectifier	Steel	5.83E-04				ecoinvent US default			56	44	0
	Aluminum	9.72E-05	1.11E-05	3.04E-05	3.00E-05	ecoinvent US default			44	56	0
	Copper	1.94E-04				0.33	0.33	0	68	32	0
	Tube insulation	9.72E-05				0.72	0.25	0	0	100	0
Hydrogen dryer and deoxidizer	Steel	9.03E-04				ecoinvent US default			56	44	0
	Glass fiber	4.52E-04				0.42	0.12	0	0	100	0
Pumps	Cast iron	1.13E-04				ecoinvent US default			56	44	0
	Steel	2.03E-04				ecoinvent US default			56	44	0
Tubing and cables	Steel	9.03E-04				ecoinvent US default			56	44	0
	Copper	3.61E-04				0.33	0.33	0	68	32	0
	Tube insulation	9.03E-05				0.72	0.25	0	0	100	0
Heat exchanger	Steel	1.13E-03				ecoinvent US default			56	44	0
Gas separators	Steel	7.78E-05				ecoinvent US default			56	44	0
Steel tank for KOH	Steel	8.27E-04				ecoinvent US default			56	44	0
Alkali-resistant pump	Polypropylene	2.92E-06				0.72	0.25	0	9	76	15
Base	Concrete	1.08E-02	6.19E-04	1.70E-03	1.68E-03	0.42	0.12	0	0	100	0
Total steel for different sub-components			7.40E-04	2.04E-03		ecoinvent US default			44	56	0
Total copper for different sub-components			3.31E-05	3.04E-05		0.33	0.33	0	68	32	0
Total plastics for different sub-components				9.12E-05		0.72	0.25	0	9	91	0

Table S4 Material weights and resource usage values used for Monte Carlo simulation of manufacturing and operation of stacks.

Life cycle stage	Sub-component	Material/ Resource/ process	Unit	Usage (Unit/kg H_2)							
				Baseline design				Advanced design			
				Minimum	*Median*	*Maximum*	*Distribution*	*Minimum*	*Median*	*Maximum*	*Distribution*
Raw materials acquisition and system manufacturing	Cathode, Anode, and Frame	Steel	kg	6.600E-03		2.160E-02	Uniform				
		Nickel	kg	1.026E-04		2.805E-03	Uniform	5.490E-04		7.008E-4	Uniform
		Aluminum	kg	3.230E-05		6.020E-05	Uniform		2.047E-05		None
		Copper	kg	2.361E-04		2.680E-04	Uniform		7.347E-06		None
		Glass fiber reinforced plastic	kg					6.929E-05		1.114E-04	Uniform
		Polypropylene	kg						7.003E-06		None
	Diaphragm, Zirfon	Zirconium oxide	kg	7.100E-05		1.430E-04	Uniform		1.285E-05		None
		Polyphenylene sulfide	kg	3.470E-05		4.760E-05	Uniform		4.284E-06		None
		Polysulfone	kg	2.650E-05		4.740E-05	Uniform		2.856E-06		None
	Gasket	Polyethylene	kg	2.560E-05		7.960E-05	Uniform		5.097E-05		None
		Tetrafluoroethylene	kg	8.000E-06		1.402E-04	Uniform	3.345E-05		5.767E-05	Uniform
		Graphite	kg	4.390E-05		5.760E-05	Uniform		4.914E-05		None
		Acrylonitrile-butadiene-styrene copolymer	kg	1.630E-05		2.140E-05	Uniform		1.827E-05		None
	Bipolar plate and End plate	Steel	kg	4.048E-02		4.521E-02	Uniform	4.355E-03		4.786E-03	Uniform
		Nickel	kg	1.464E-03		1.470E-03	Uniform	1.316E-04		1.680E-04	Uniform
	Manufacturing	Electricity	kWh		1.940E-02		None		7.700E-03		None
Operation and maintenance	Stack	Electricity	kWh	53.60		58.75	Uniform	45.56		48.56	Uniform
		Liquid nitrogen	kg		2.900E-04		None		2.900E-04		None
		Potassium hydroxide	kg	8.500E-04		1.900E-03	Uniform	8.500E-04		1.900E-03	Uniform
		Deionized water	kg	10		30	Uniform	10		30	Uniform

Note: The end-of-life treatment stage of the life cycle included all the materials in the raw materials acquisition and system manufacturing stage defined in this table where these weights were proportioned into the recycling, landfilling, and incineration rates defined in Table S2.

Table S5 Materials weights and resource usage values used for Monte Carlo simulation of manufacturing and operation of balance of plant.

Life cycle stages	Sub-component	Material/Resource/Process	Unit	Usage (Unit/kg H_2)			
				Minimum	Median	Maximum	Distribution
Raw materials acquisition and system manufacturing	Water purifier and feed tank	Steel	kg		4.500E-04		None
		Polyethylene	kg		2.300E-04		None
	Control panel	Electronics	kg		9.720E-05		None
	Transformer and rectifier	Steel	kg		5.830E-04		None
		Aluminum	kg		9.720E-05		None
		Copper	kg		1.940E-04		None
		Tube insulation	kg		9.720E-05		None
	Hydrogen dryer and deoxidizer	Steel	kg		4.520E-04		None
		Glass fiber	kg		9.030E-04		None
	Pumps	Cast iron	kg		1.130E-04		None
		Steel	kg		2.030E-04		None
	Tubing and cables	Steel	kg		9.030E-04		None
		Copper	kg		3.610E-05		None
		Tube insulation	kg		9.000E-05		None
	Heat exchanger	Steel	kg		1.130E-03		None
	Gas separators	Steel	kg		7.780E-05		None
	Steel tank for KOH	Steel	kg		8.270E-04		None
	Alkali-resistant pump	Polypropylene	kg		2.920E-06		None
	Base	Concrete	kg	6.190E-04		1.078E-02	Uniform
	Manufacturing	Steel sheet rolling	kg	7.400E-04		4.853E-03	Uniform
		Aluminum sheet rolling	kg	1.110E-05		9.721E-05	Uniform
		Copper wire drawing	kg	3.040E-05		5.558E-04	Uniform
		Plastic injection molding	kg	9.120E-05		4.546E-04	Uniform
Operation and maintenance	Baseline stack design	Electricity	kWh		5.5		None
	Advanced stack design	Electricity	kWh		3.8		None

Note: The end-of-life treatment stage of the life cycle included all the materials in the raw materials acquisition and system manufacturing stage defined in this table where these weights were proportioned into the recycling, landfilling, and incineration rates defined in Table S3.

Table S6 Overall life cycle environmental impacts of a LAWE to produce 1 kg H_2 across various scenarios.

**Categories**	**Scenarios**								
Electricity source	*Solar*			*Hybrid*			*Wind*		
Stack design	*Baseline*	*Advanced*		*Baseline*	*Advanced*		*Baseline*	*Advanced*	
Stack end-of-life	*No recycling*		*Recycling*	*No recycling*		*Recycling*	*No recycling*		*Recycling*
Climate change (kg CO_2-eq)	3.27-5.09	2.64-4.09	2.57-4.15	2.25-3.41	1.74-2.70	1.76-2.57	1.37-2.18	1.05-1.64	1.03-1.68
Acidification (kg SO_2-eq)	0.018-0.027	0.013-0.02	0.013-0.02	0.016-0.023	0.012-0.017	0.012-0.016	0.014-0.019	0.01-0.014	0.009-0.013
Eutrophication (kg N-eq)	0.013-0.029	0.01-0.023	0.01-0.023	0.011-0.022	0.009-0.018	0.009-0.017	0.009-0.016	0.007-0.013	0.006-0.013
Ozone depletion (kg CFC-11-eq)	1.93E-07-7.09E-07	3.14E-07-4.83E-07	3.1E-07-4.88E-07	7.76E-08-5.86E-07	2.27E-07-3.74E-07	2.22E-07-3.78E-07	6.8E-08-3.57E-07	1.38E-07-2.66E-07	1.34E-07-2.69E-07
Eco-toxicity (CTUe)	173-362	141-276	137-277	514-754	414-608	411-609	814-1188	663-965	655-970
Human toxicity (CTUh)	1.5E-06-5.1E-06	1.16E-06-3.77E-06	1.08E-06-3.82E-06	1.78E-06-5.6E-06	1.3E-06-4.27E-06	1.4E-06-4.14E-06	2.06E-06-6.1E-06	1.61E-06-4.6E-06	1.48E-06-4.7E-06
PM formation (kg PM2.5-eq)	0.0033-0.0054	0.0025-0.0042	0.0025-0.0042	0.0028-0.0041	0.0021-0.0032	0.0021-0.0031	0.002-0.0031	0.0015-0.0023	0.0014-0.0023
Smog formation (kg O_3-eq)	0.20-0.30	0.15-0.24	0.15-0.24	0.15-0.22	0.12-0.18	0.12-0.17	0.10-0.16	0.08-0.12	0.08-0.12
Fossil energy use (kg oil-eq)	0.74-1.16	0.61-0.92	0.61-0.91	0.55-0.77	0.43-0.60	0.43-0.60	0.29-0.43	0.21-0.33	0.22-0.33
Land use (m^2·a crop)	0.63-1.68	0.54-1.36	0.52-1.38	0.35-0.88	0.30-0.72	0.29-0.72	0.06-0.10	0.05-0.08	0.05-0.08
Materials depletion (kg Cu-eq)	0.13-0.18	0.08-0.11	0.08-0.11	0.14-0.19	0.09-0.12	0.09-0.12	0.15-0.20	0.09-0.14	0.09-0.13
Water use (m^3)	0.104-0.16	0.082-0.124	0.081-0.123	0.062-0.092	0.046-0.069	0.046-0.067	0.017-0.027	0.010-0.015	0.009-0.014

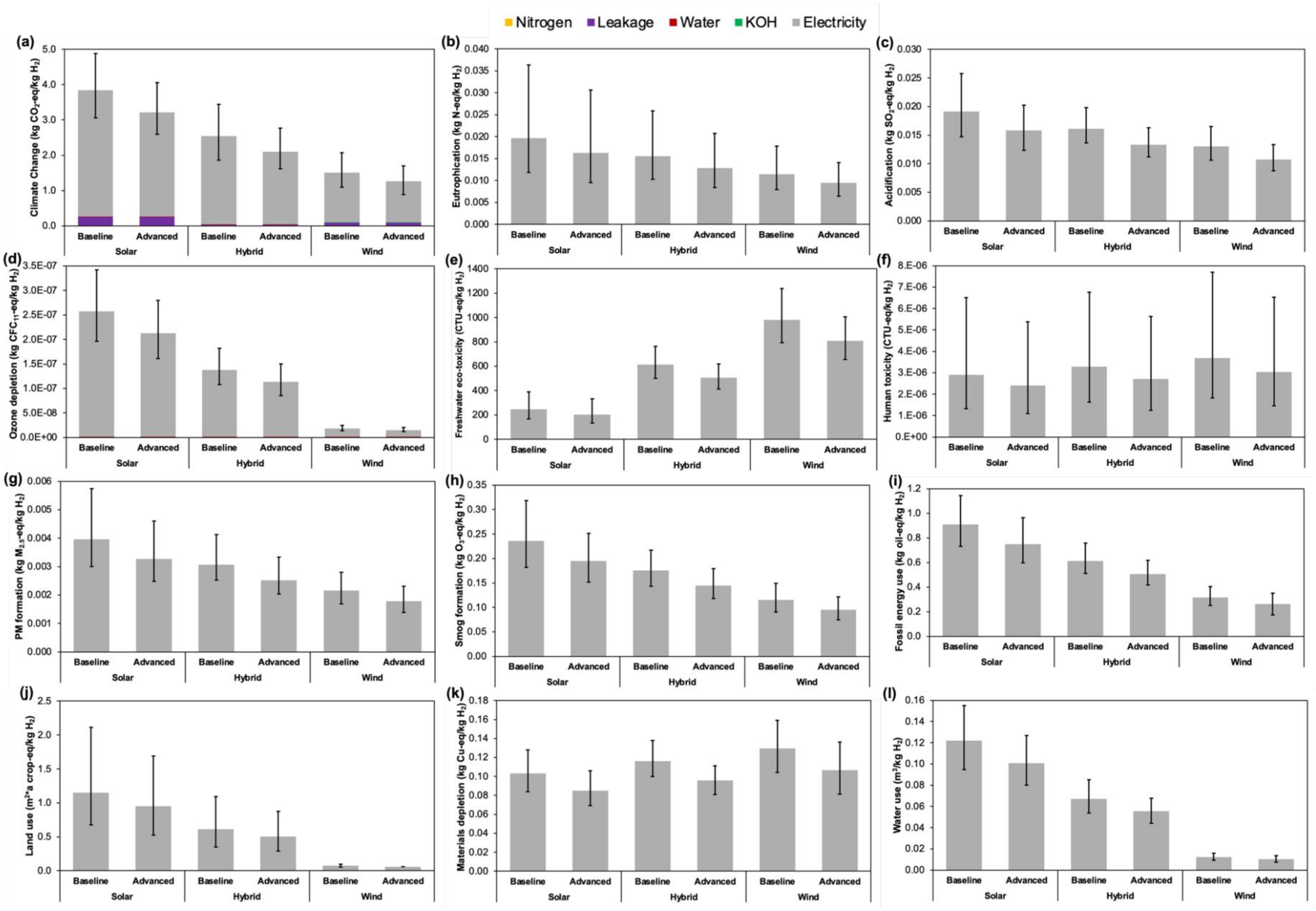

Figure S1 Breakdown of the 12 impact categories based on the different contributors of the operation and maintenance stage of the life cycle: **(a)** climate change, **(b)** eutrophication, **(c)** acidification, **(d)** ozone depletion potential, **(e)** freshwater eco-toxicity, **(f)** carcinogenic human toxicity, **(g)** particulate matter (PM) formation, **(h)** photochemical smog formation, **(i)** fossil energy use, **(j)** land use, **(k)** materials depletion, and **(l)** water use. Error bars represent uncertainty ranges from Monte Carlo simulations.

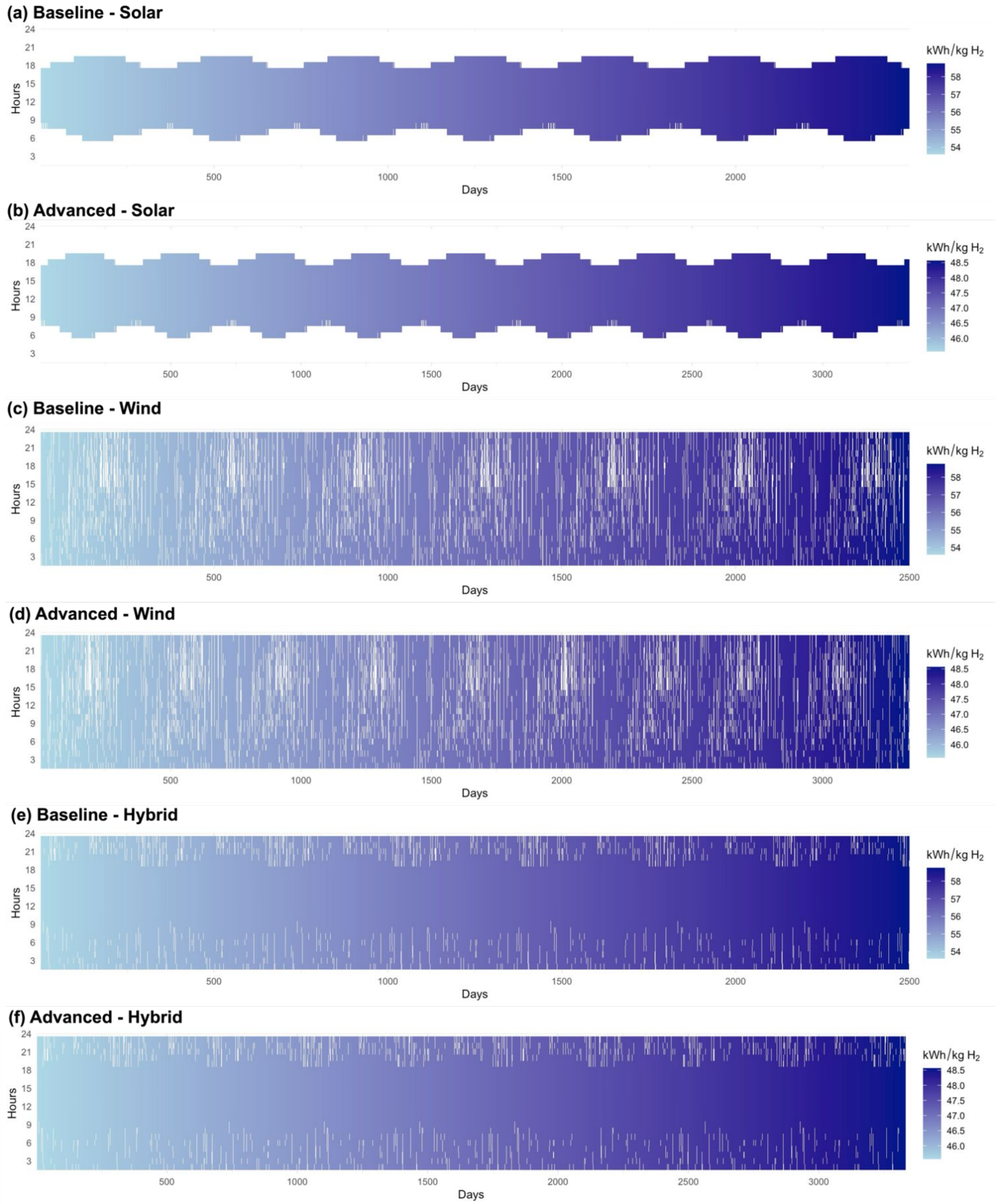

Figure S2 Hourly stack energy use over their lifetimes: **(a)** baseline stack connected to solar panels, **(b)** advanced stack connected to solar panels, **(c)** baseline stack connected to wind turbines, **(d)** advanced stack connected to wind turbines, **(e)** baseline stack connected to hybrid electicity source, and **(f)** advanced stack connected to hybrid electricity source.

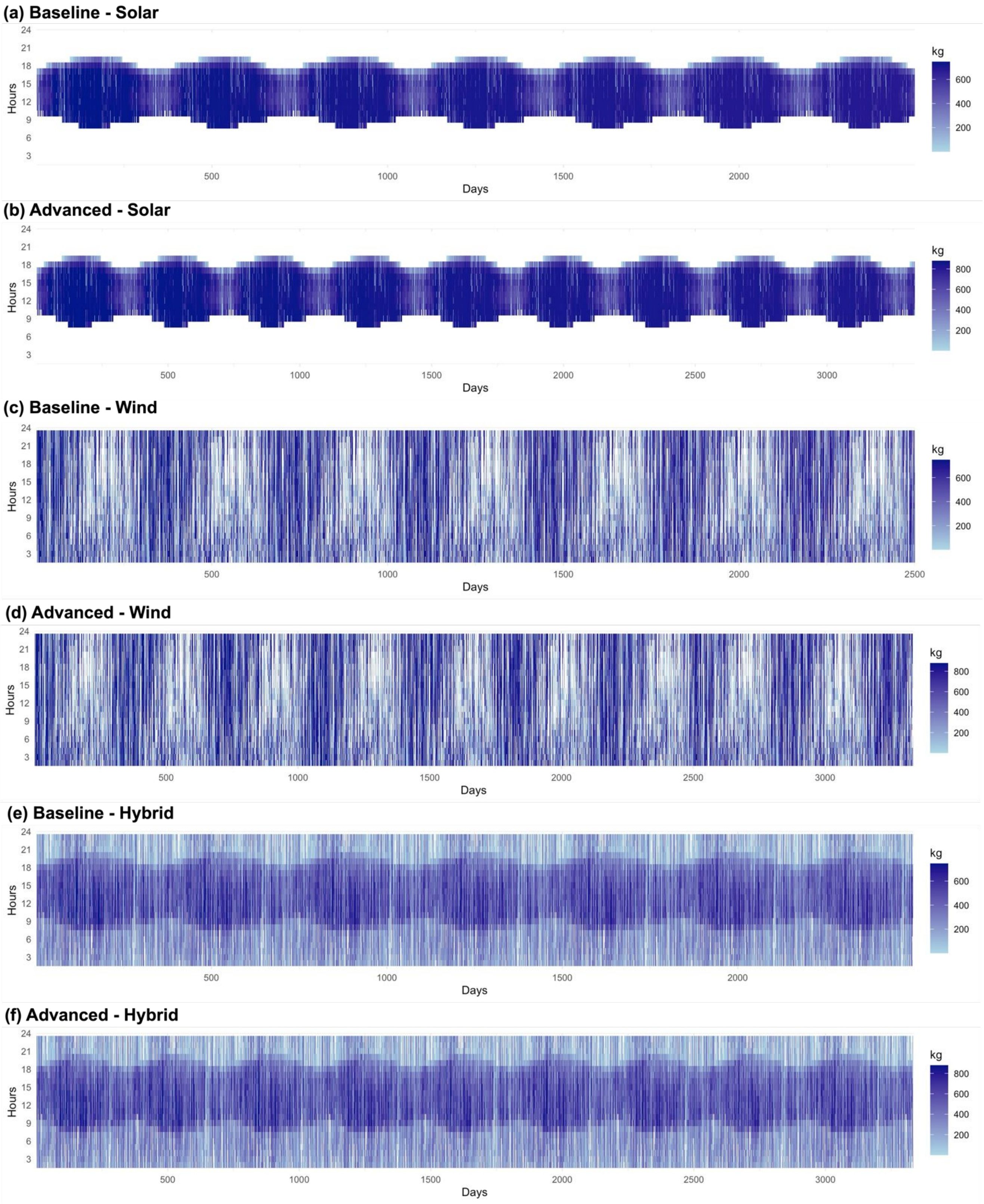

Figure S3 Hourly hydrogen production over stack lifetimes: **(a)** baseline stack connected to solar panels, **(b)** advanced stack connected to solar panels, **(c)** baseline stack connected to wind turbines, **(d)** advanced stack connected to wind turbines, **(e)** baseline stack connected to hybrid electicity source, and **(f)** advanced stack connected to hybrid electricity source.

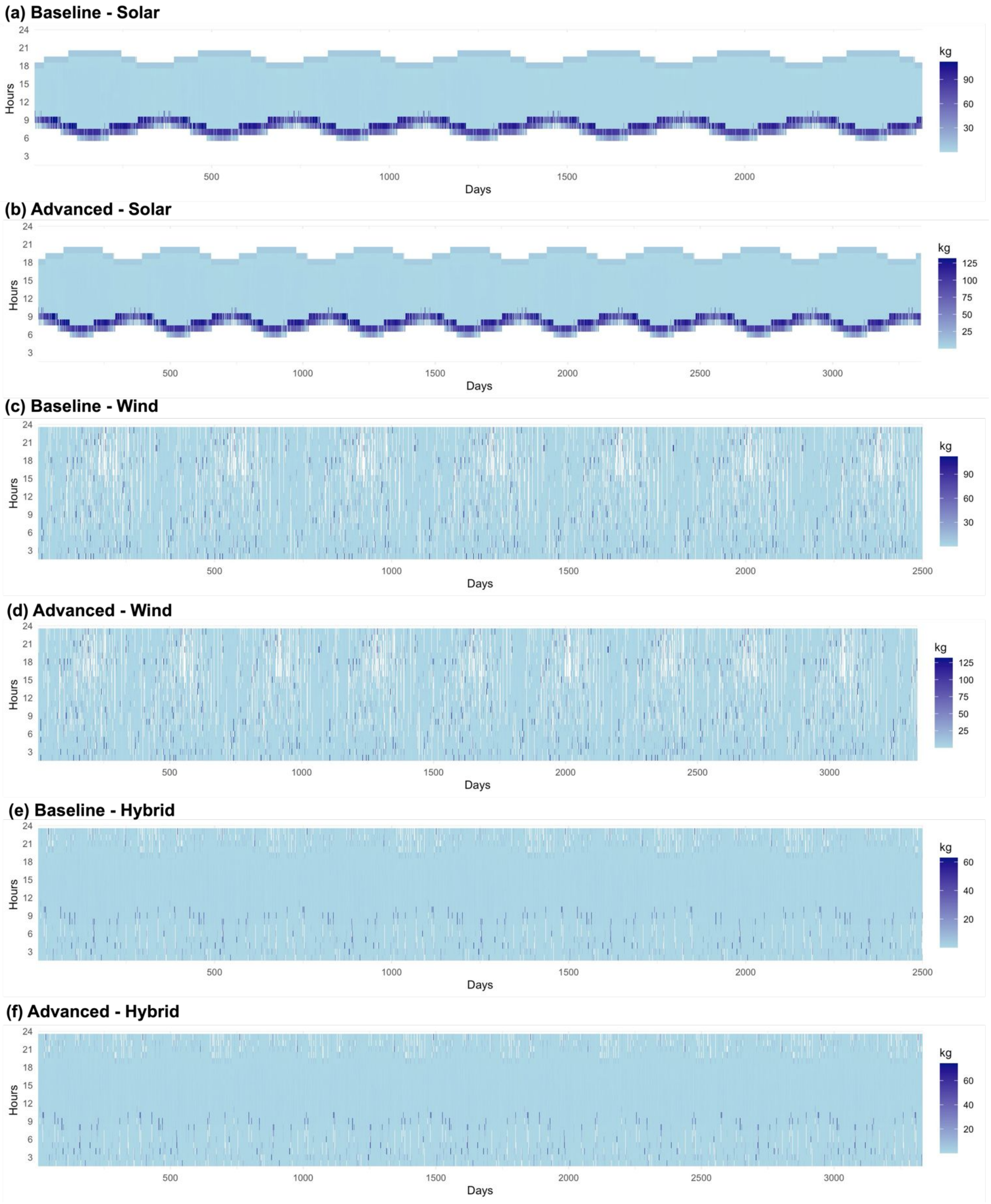

Figure S4 Hourly hydrogen leakage over stack lifetimes: **(a)** baseline stack connected to solar panels, **(b)** advanced stack connected to solar panels, **(c)** baseline stack connected to wind turbines, **(d)** advanced stack connected to wind turbines, **(e)** baseline stack connected to hybrid electicity source, and **(f)** advanced stack connected to hybrid electricity source.

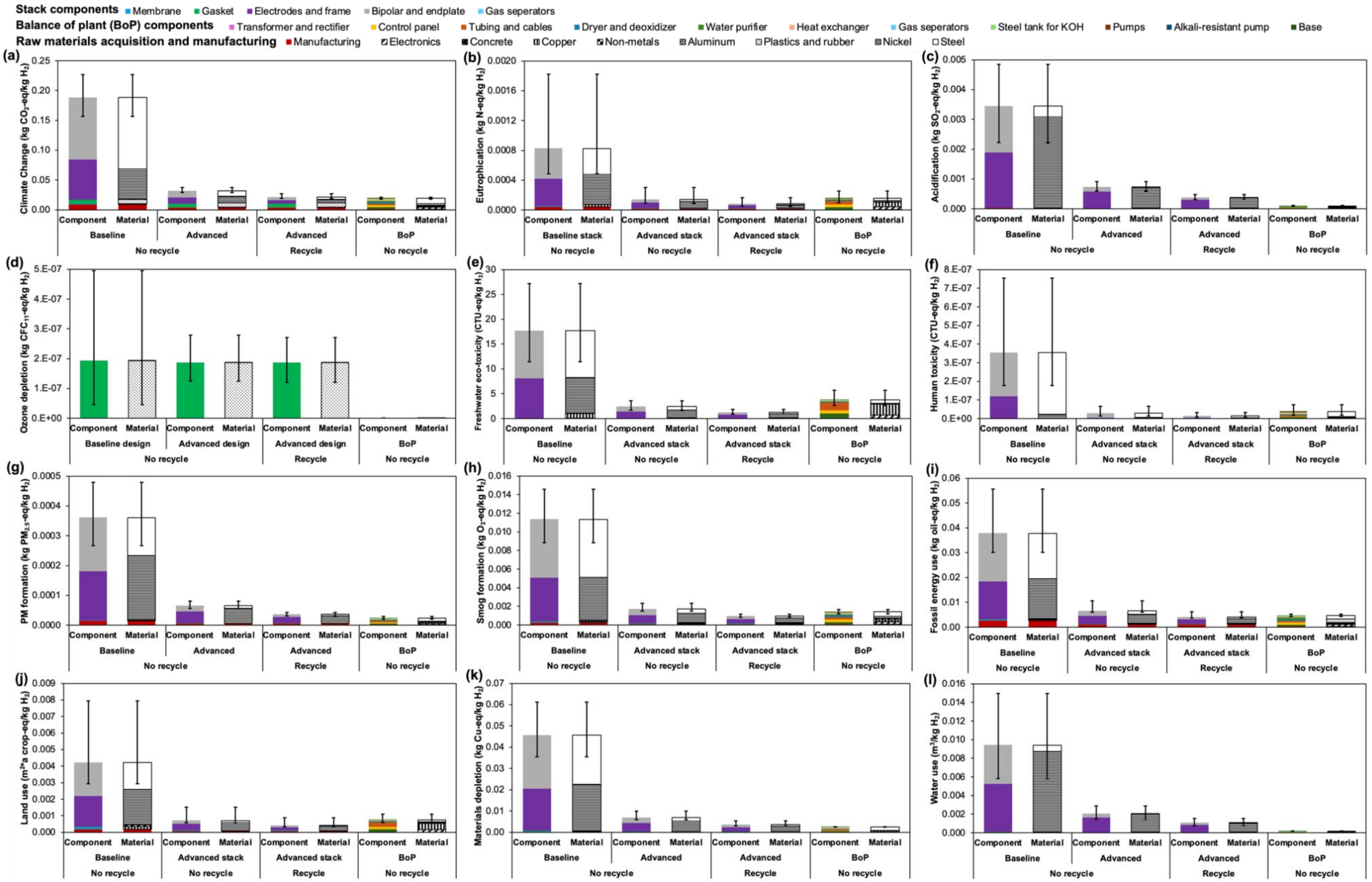

Figure S5 Breakdown of the 12 embodied impact categories based on the different components and materials of stacks and balance of plant: **(a)** climate change, **(b)** eutrophication, **(c)** acidification, **(d)** ozone depletion potential, **(e)** freshwater eco-toxicity, **(f)** carcinogenic human toxicity, **(g)** particulate matter (PM) formation, **(h)** photochemical smog formation, **(i)** fossil energy use, **(j)** land use, **(k)** materials depletion, and **(l)** water use. Error bars represent uncertainty ranges from Monte Carlo simulations.

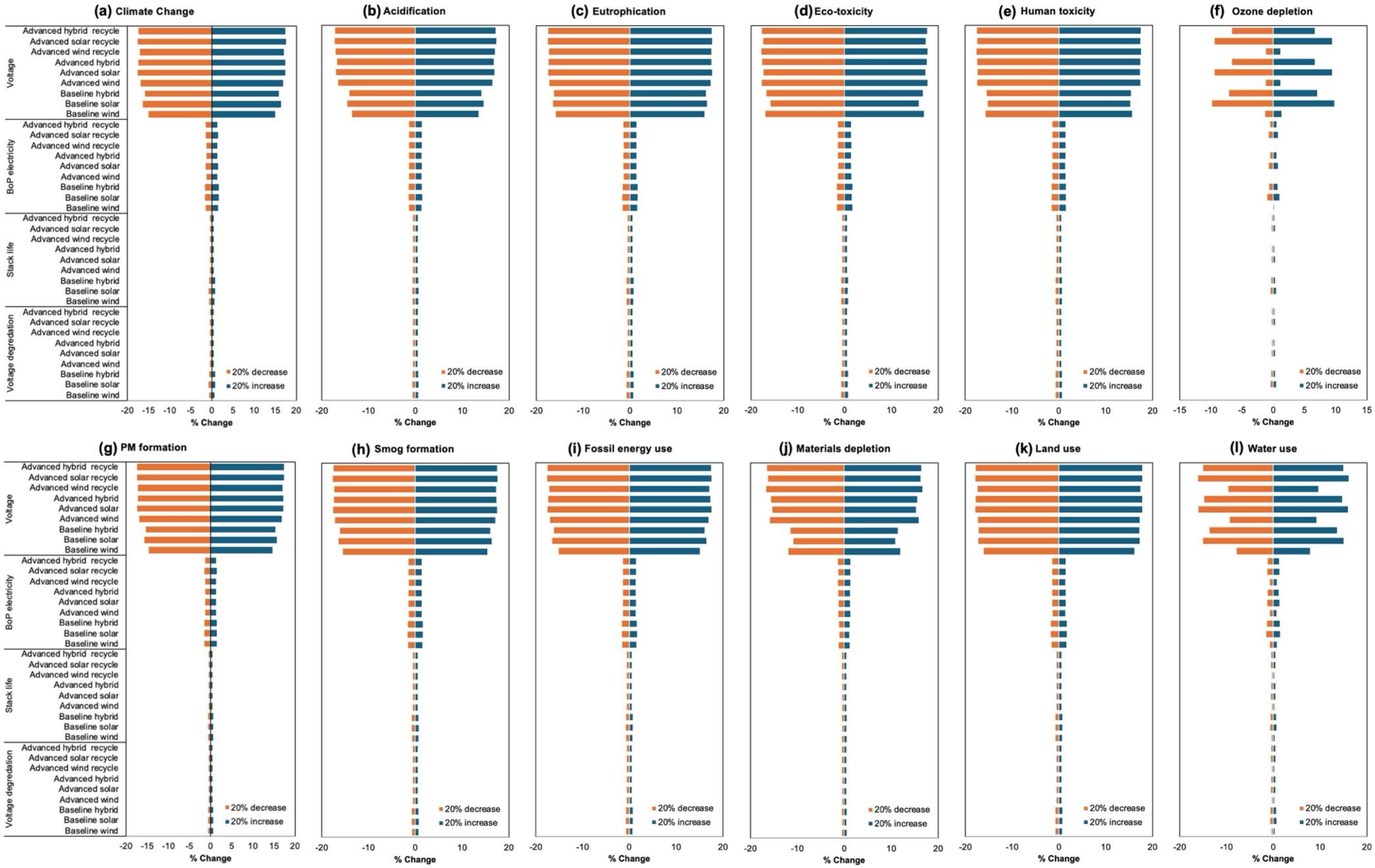

Figure S6 Tornado plots showing the results of the ±20% parameter sensitivity analysis for **(a)** climate change, **(b)** acidification, **(c)** eutrophication, **(d)** freshwater eco-toxicity, **(e)** carcinogenic human toxicity, **(f)** ozone depletion, **(g)** particulate matter (PM) formation, **(h)** smog formation, **(i)** fossil energy use, **(j)** materials depletion, **(k)** land use, and **(l)** water use.

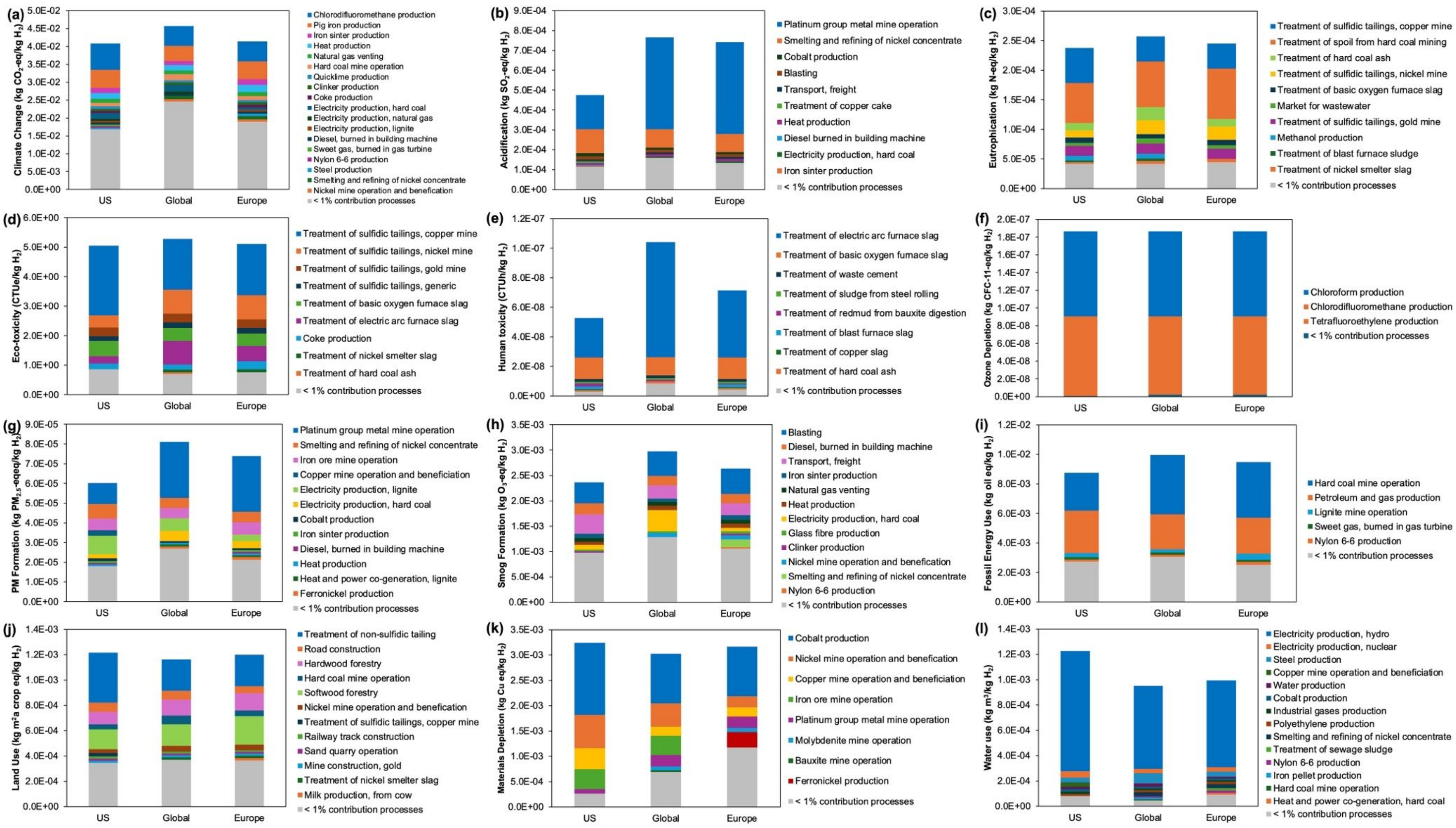

Figure S7 Contribution analysis of manufacturing advanced stack with recycling using US-representative, global, and European inventories for **(a)** climate change, **(b)** acidification, **(c)** eutrophication, **(d)** freshwater eco-toxicity, **(e)** carcinogenic human toxicity, **(f)** ozone depletion, **(g)** particulate matter (PM) formation, **(h)** smog formation, **(i)** fossil energy use, **(j)** materials depletion, **(k)** land use, and **(l)** water use.

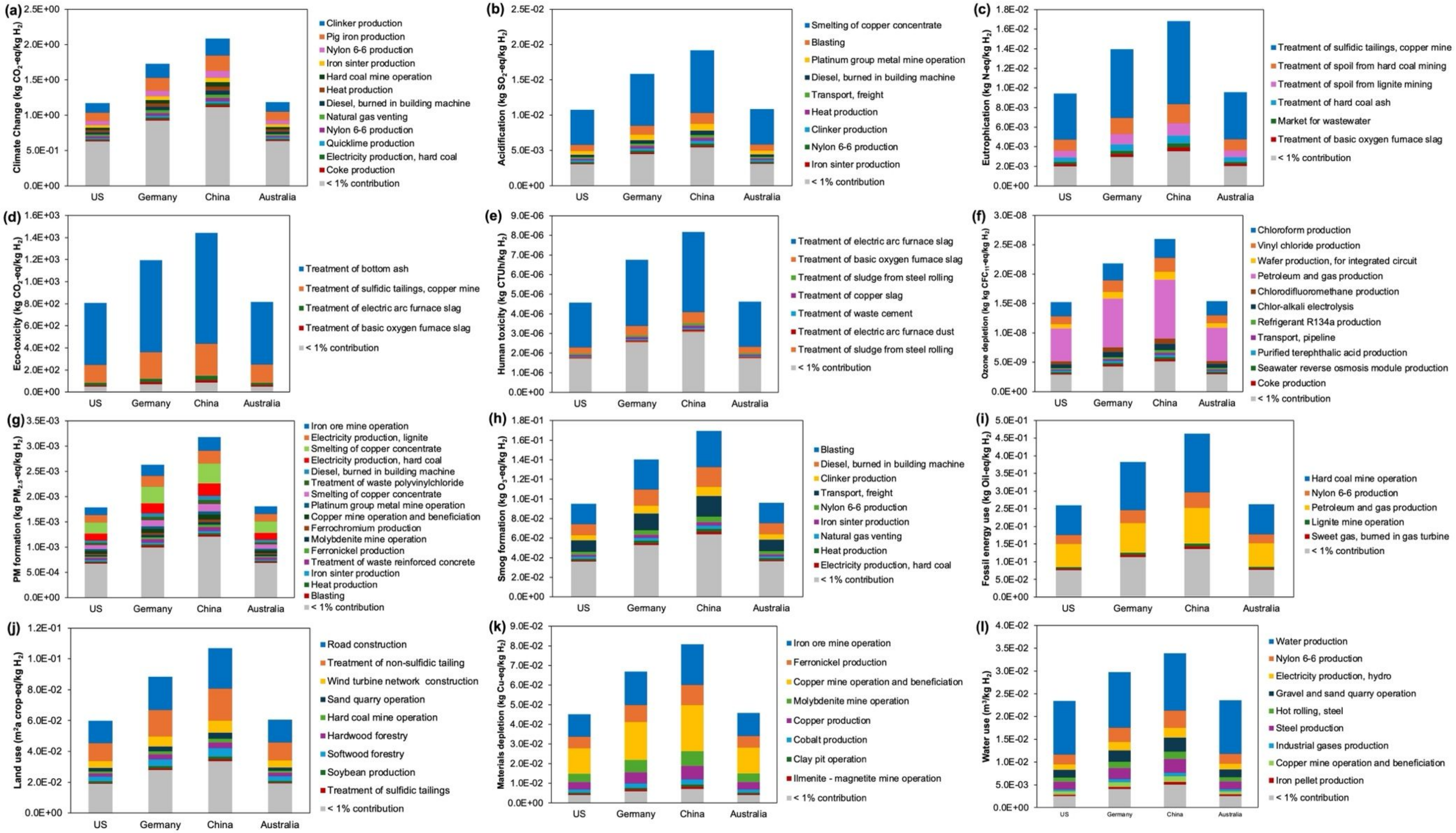

Figure S7 Contribution analysis of O&M of advanced using US-representative, German, Chinese, and Australian inventories for **(a)** climate change, **(b)** acidification, **(c)** eutrophication, **(d)** freshwater eco-toxicity, **(e)** carcinogenic human toxicity, **(f)** ozone depletion, **(g)** particulate matter (PM) formation, **(h)** smog formation, **(i)** fossil energy use, **(j)** materials depletion, **(k)** land use, and **(l)** water use.

References

(1) Iyer, R. K.; Prosser, J. H.; Kelly, J. C.; James, B. D.; Elgowainy, A. Life-Cycle Analysis of Hydrogen Production from Water Electrolyzers. *International Journal of Hydrogen Energy* **2024**, *81*, 1467–1478. https://doi.org/10.1016/j.ijhydene.2024.06.355.

(2) Koj, J. C.; Wulf, C.; Schreiber, A.; Zapp, P. Site-Dependent Environmental Impacts of Industrial Hydrogen Production by Alkaline Water Electrolysis. *Energies* **2017**, *10* (7), 860. https://doi.org/10.3390/en10070860.

(3) Lim, D.; Lee, B.; Lee, H.; Byun, M.; Cho, H.-S.; Cho, W.; Kim, C.-H.; Brigljević, B.; Lim, H. Impact of Voltage Degradation in Water Electrolyzers on Sustainability of Synthetic Natural Gas Production: Energy, Economic, and Environmental Analysis. *Energy Conversion and Management* **2021**, *245*, 114516. https://doi.org/10.1016/j.enconman.2021.114516.

(4) Kaur, A. Hydrogen Emissions from an Electrolysis Unit. Master of Science in Sustainable Energy Technology, Delft University of Technology, Delft, Netherlands, 2023. https://repository.tudelft.nl/file/File_209db7cf-1093-47bd-b030-debbb96f0290?preview=1.

(5) Ocko, I. B.; Hamburg, S. P. Climate Consequences of Hydrogen Emissions. *Atmospheric Chemistry and Physics* **2022**, *22* (14), 9349–9368. https://doi.org/10.5194/acp-22-9349-2022.

(6) Sand, M.; Skeie, R. B.; Sandstad, M.; Krishnan, S.; Myhre, G.; Bryant, H.; Derwent, R.; Hauglustaine, D.; Paulot, F.; Prather, M.; Stevenson, D. A Multi-Model Assessment of the Global Warming Potential of Hydrogen. *Commun Earth Environ* **2023**, *4* (1), 1–12. https://doi.org/10.1038/s43247-023-00857-8.

(7) Wulf, C.; Kaltschmitt, M. Hydrogen Supply Chains for Mobility—Environmental and Economic Assessment. *Sustainability* **2018**, *10* (6), 1699. https://doi.org/10.3390/su10061699.

(8) Lee, B.; Cho, H.-S.; Kim, H.; Lim, D.; Cho, W.; Kim, C.-H.; Lim, H. Integrative Techno-Economic and Environmental Assessment for Green H2 Production by Alkaline Water Electrolysis Based on Experimental Data. *Journal of Environmental Chemical Engineering* **2021**, *9* (6), 106349. https://doi.org/10.1016/j.jece.2021.106349.

(9) Gerloff, N. Comparative Life-Cycle-Assessment Analysis of Three Major Water Electrolysis Technologies While Applying Various Energy Scenarios for a Greener Hydrogen Production. *Journal of Energy Storage* **2021**, *43*, 102759. https://doi.org/10.1016/j.est.2021.102759.

(10) Lotrič, A.; Sekavčnik, M.; Kuštrin, I.; Mori, M. Life-Cycle Assessment of Hydrogen Technologies with the Focus on EU Critical Raw Materials and End-of-Life Strategies. *International Journal of Hydrogen Energy* **2021**, *46* (16), 10143–10160. https://doi.org/10.1016/j.ijhydene.2020.06.190.

(11) Zhang, J.; Wang, Z.; He, Y.; Li, M.; Wang, X.; Wang, B.; Zhu, Y.; Cen, K. Comparison of Onshore/Offshore Wind Power Hydrogen Production through Water Electrolysis by Life Cycle Assessment. *Sustainable Energy Technologies and Assessments* **2023**, *60*, 103515. https://doi.org/10.1016/j.seta.2023.103515.

(12) Krishnan, S.; Corona, B.; Kramer, G. J.; Junginger, M.; Koning, V. Prospective LCA of Alkaline and PEM Electrolyser Systems. *International Journal of Hydrogen Energy* **2024**, *55*, 26–41. https://doi.org/10.1016/j.ijhydene.2023.10.192.

(13) Acevedo, Y. M.; Prosser, J. H.; Huya-Kouadio, J. M.; McNamara, K. R.; James, B. D. *Hydrogen Production Cost with Alkaline Electrolysis*; DOE-SA-09629-1; Strategic Analysis, Inc., Arlington VA (United States), 2023. https://doi.org/10.2172/2203367.

(14) Hwang, H.-L.; Lim, H.; Chin, S.-M.; Uddin, M.; Biehl, A.; Xie, F.; Hargrove, S.; Liu, Y.; Wang, R. *Freight Analysis Framework Version 5 (FAF5) Base Year 2017 Data Development Technical Report*; ORNL/TM-2021/2154; Oak Ridge National Laboratory (ORNL), Oak Ridge, TN (United States), 2021. https://doi.org/10.2172/1844893.

(15) US EPA. *Plastics: Material-Specific Data*. United States Environmental Protection Agency (US EPA). https://www.epa.gov/facts-and-figures-about-materials-waste-and-recycling/plastics-material-specific-data (accessed 2024-10-14).

(16) USGS. *Minerals Yearbook - Metals and Minerals*. U.S. Geological Survey. https://www.usgs.gov/centers/national-minerals-information-center/minerals-yearbook-metals-and-minerals#S (accessed 2024-10-14).
